# The small molecule activator S3969 stimulates the epithelial sodium channel by interacting with a specific binding pocket in the channel's β-subunit

**DOI:** 10.1016/j.jbc.2024.105785

**Published:** 2024-02-23

**Authors:** Florian Sure, Jürgen Einsiedel, Peter Gmeiner, Patrick Duchstein, Dirk Zahn, Christoph Korbmacher, Alexandr V. Ilyaskin

**Affiliations:** 1 Institute of Cellular and Molecular Physiology, Friedrich-Alexander-Universität Erlangen-Nürnberg, Erlangen, Germany; 2 Department of Chemistry and Pharmacy, Friedrich-Alexander-Universität Erlangen-Nürnberg, Erlangen, Germany; 3 Theoretical Chemistry/Computer Chemistry Center (CCC), Friedrich-Alexander-Universität Erlangen-Nürnberg, Erlangen, Germany

**Keywords:** epithelial sodium channel (ENaC), electrophysiology, two electrode voltage clamp, molecular docking, molecular dynamics, site-directed mutagenesis, small molecule activator, S3969, oocyte, H441 cell line

## Abstract

The epithelial sodium channel (ENaC) is essential for mediating sodium absorption in several epithelia. Its impaired function leads to severe disorders, including pseudohypoaldosteronism type 1 and respiratory distress. Therefore, pharmacological ENaC activators have potential therapeutic implications. Previously, a small molecule ENaC activator (S3969) was developed. So far, little is known about molecular mechanisms involved in S3969-mediated ENaC stimulation. Here, we identified an S3969-binding site in human ENaC by combining structure-based simulations with molecular biological methods and electrophysiological measurements of ENaC heterologously expressed in *Xenopus laevis* oocytes. We confirmed a previous observation that the extracellular loop of β-ENaC is essential for ENaC stimulation by S3969. Molecular dynamics simulations predicted critical residues in the thumb domain of β-ENaC (Arg388, Phe391, and Tyr406) that coordinate S3969 within a binding site localized at the β-γ-subunit interface. Importantly, mutating each of these residues reduced (R388H; R388A) or nearly abolished (F391G; Y406A) the S3969-mediated ENaC activation. Molecular dynamics simulations also suggested that S3969-mediated ENaC stimulation involved a movement of the α5 helix of the thumb domain of β-ENaC away from the palm domain of γ-ENaC. Consistent with this, the introduction of two cysteine residues (βR437C – γS298C) to form a disulfide bridge connecting these two domains prevented ENaC stimulation by S3969 unless the disulfide bond was reduced by DTT. Finally, we demonstrated that S3969 stimulated ENaC endogenously expressed in cultured human airway epithelial cells (H441). These new findings may lead to novel (patho-)physiological and therapeutic concepts for disorders associated with altered ENaC function.

The epithelial sodium channel (ENaC) is a heterotrimeric ion channel, which consists of α, β, and γ subunits and belongs to the ENaC/degenerin (DEG) family of ion channels (1). In several vertebrate species, including humans, an additional δ-subunit can functionally replace the α-subunit, altering ENaC function and regulation (2, 3, 4). Each ENaC subunit comprises short intracellular N- and C-termini, two transmembrane domains, and a large extracellular loop (ECL) (5, 6). Recently, the molecular 3D structure of the ECLs in human αβγ-ENaC was resolved using cryo-EM (7, 8). The ECL structure of an individual ENaC subunit resembles a hand holding a ball with palm, finger, thumb, knuckle, and β-ball domains. ENaC shares this overall ECL structure with other members of the ENaC/DEG family of ion channels including, for example, acid sensing ion channel (ASIC) and FMRFamide-gated sodium channel (FaNaC) (9, 10). In addition, a unique feature of ENaC is its gating relief of inhibition (GRIP) domain which forms an extension of the finger domain and is involved in proteolytic channel activation (7, 8, 11, 12, 13, 14).

ENaC provides the rate-limiting pathway for apical sodium entry into epithelial cells and is critical for transepithelial sodium absorption in several epithelia, including the distal nephron, distal colon, lung epithelia, sweat, and salivary ducts. In particular, the appropriate regulation of ENaC function is essential for maintaining sodium homeostasis, blood pressure control, and pulmonary fluid balance (15, 16, 17, 18, 19). This becomes evident in disease states when ENaC function is deranged. Increased ENaC activity in the distal nephron is likely to contribute to the pathophysiology of essential hypertension, in particular in a subset of patients with salt-sensitive hypertension (18, 20). In the lungs, hyperactive ENaC probably plays a role in the development of cystic fibrosis (21, 22, 23). Conversely, loss-of-function ENaC mutations can result in life-threatening pseudohypoaldosteronism type 1B (PHA1B), with severe hypotension and renal salt wasting (24, 25, 26, 27). Moreover, ENaC dysfunction in the lungs is linked to impaired pulmonary fluid clearance, contributing to respiratory distress and pulmonary edema (17, 28, 29, 30, 31, 32).

Pharmacological stimulation of ENaC may represent a promising therapeutic concept for diseases associated with reduced ENaC activity. However, only a few ENaC activators have been developed to date. The best-studied example is AP301 (solnatide), a peptidomimetic drug resembling the lectin-like domain of the cytokine tumor necrosis factor, which stimulates ENaC probably through direct interaction with its α-subunit (33, 34, 35, 36, 37). AP301 (or the tumor necrosis factor lectin-like domain) was shown to stimulate ENaC carrying PHA1B mutations (38), to improve pulmonary fluid clearance and ameliorate respiratory distress in animal models (39, 40, 41), and is currently evaluated in clinical trials (42). In 2008, a small molecule ENaC activator S3969 was described (43). S3969 is a tripeptide-like substance that activated human but not mouse αβγ-ENaC in low micromolar concentrations in heterologous expression systems (43). Importantly, S3969 stimulated mutant channels carrying a PHA1B mutation (β_G37S_) (43) or a partial loss-of-function mutation associated with atypical cystic fibrosis (α_F61L_) (44). Initial characterization of S3969 revealed that its stimulatory effect critically depends on the ECL of the β-ENaC subunit (43). However, the underlying molecular mechanisms, including the precise localization of the S3969 binding site in β-ENaC, remain unknown.

In this study, we used a chimeric approach and the available structural information on ENaC to identify and characterize the S3969-binding site in the channel's β-subunit. Moreover, using atomistic molecular dynamics (MD) simulations, we attempted to shed light on the molecular mechanisms involved in ENaC activation by S3969. Predictions from MD simulations were tested using site-directed mutagenesis and electrophysiological studies of the generated ENaC mutants. In addition, we performed proof of principle experiments to demonstrate the effect of S3969 on ENaC-mediated transport in H441 human distal airway epithelial cells.

## Results

### Replacing the extracellular loop of mouse β-ENaC with that of human β-ENaC converted mouse ENaC into an S3969-sensitive ion channel

**Figure 1:**
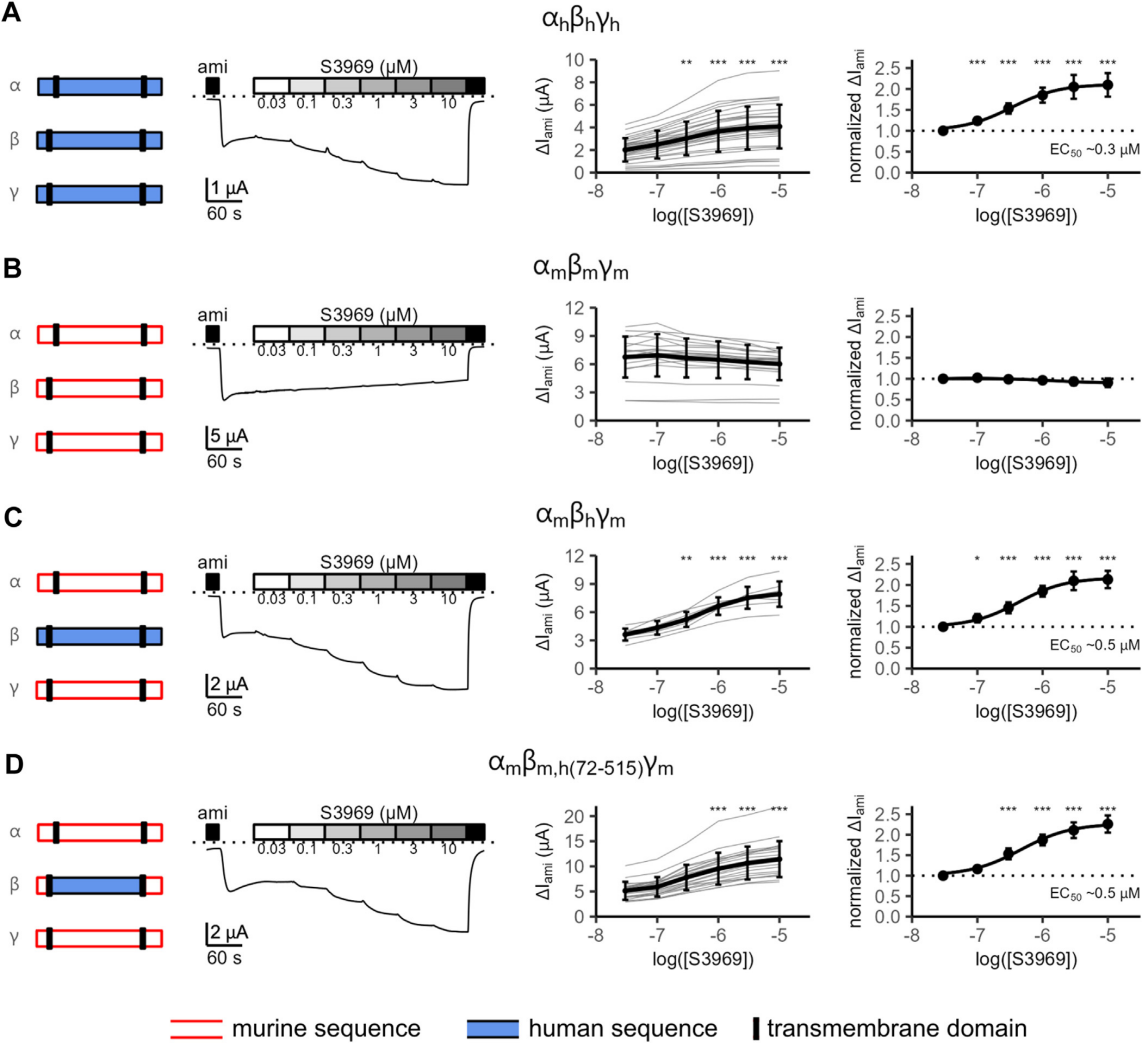
**S3969 activates human but not mouse ENaC, and the ECL of human β-ENaC is essential for this stimulatory effect.***A*–*D*, *first panels*: Pictograms symbolize α-, β-, and γ-ENaC subunits, which were co-expressed in the corresponding group of oocytes. Murine or human isoforms and parts of the chimeric β-ENaC are shown in *white* with a *red* outline or in *blue* with a *black* outline, respectively. Transmembrane domains are represented as *black* bars. *Second panels*: A representative whole-cell current trace is shown for an oocyte expressing ENaC with the subunit composition as indicated in the corresponding *first panel*. Amiloride (ami, 2 μM) and S3969 (at the indicated concentration in μM) were present in the bath solution as indicated by *black* and *gray* shaded bars, respectively. *Dotted* lines correspond to the zero current level. *Third panels*: ENaC-mediated amiloride-sensitive whole-cell current values (ΔI_ami_) were determined from similar experiments as shown in the *second panel*. *Gray* lines connect data points obtained in an individual oocyte. Mean ± SD are shown in *black* (*A*: N = 7, n = 29; *B*: N = 4, n = 19; *C*: N = 2, n = 7; *D*: N = 2, n = 19; N indicates the number of different batches of oocytes, n indicates the number of individual oocytes studied per experimental group). *Fourth panels*: Concentration-response relationship of the S3969 effect on ENaC currents. In each individual recording shown in the *third panel*, ΔI_ami_ was normalized to ΔI_ami_ obtained with 0.03 μM of S3969. Mean ± SD are shown and fitted to Equation 1 (in *A*, *C*, and *D*) to estimate EC_50_, the half maximal stimulatory concentration. In (*B*), data were fitted to a spline function. *Dotted* lines indicate normalized ΔI_ami_ values of one (no effect). One-way ANOVA with Bonferroni’s post hoc test was used to calculate *p*-values for comparisons with the baseline current values: in *third panels* (*A*: 1/1/0.009/<0.0001/<0.0001/<0.0001; *B*: 1/1/1/1/1/1; *C*: 1/0.8/0.004/<0.0001/<0.0001/<0.0001; *D*: 1/1/0.106/<0.0001/<0.0001/<0.0001); in *fourth panels* (*A*: 1/<0.0001/<0.0001/<0.0001/<0.0001/<0.0001; *B*: 1/1/1/0.633/0.010/<0.0001; *C*: 1/0.030/<0.0001/<0.0001/<0.0001/<0.0001; *D*: 1/0.400/<0.0001/<0.0001/<0.0001/<0.0001). ∗*p* < 0.05, ∗∗*p* < 0.01 and ∗∗∗*p* < 0.001 indicate significant stimulation by S3969. ENaC, epithelial sodium channel.

Previously, it was demonstrated that S3969 can stimulate human but not mouse ENaC in its αβγ-subunit configuration. Moreover, it was shown that the ECL of human β-ENaC was essential to mediate this stimulatory effect (43). To confirm these findings, we heterologously expressed human or mouse α-, β-, and γ-ENaC in *Xenopus laevis* oocytes. ENaC function was assessed by measuring amiloride-sensitive inward currents (ΔI_ami_) using the two-electrode voltage clamp technique. We first performed experiments with oocytes expressing all three human ENaC subunits (α_h_β_h_γ_h_) as schematically depicted in Figure 1*A* (*first panel*). The *second panel* shows a representative continuous whole-cell current trace recorded at a holding potential of −60 mV of an oocyte expressing α_h_β_h_γ_h_-ENaC. Recordings were started in the presence of 2 μM amiloride, which inhibits human ENaC reversibly and almost completely (>90%) at this concentration (1, 43). Washout of amiloride revealed an ENaC-mediated Na^+^ inward current component. Subsequent extracellular application of S3969 at increasing concentrations ranging from 0.03 μM to 10 μM demonstrated a concentration-dependent increase of inward currents. Reapplication of amiloride at the end of the measurement returned the current to its initial level. This confirms that the inward currents stimulated by S3969 were mediated by ENaC. Results from similar experiments are summarized in the third and fourth panels of Figure 1*A* as absolute and normalized ΔI_ami_ values, respectively. On average, S3969 activated human ENaC (α_h_β_h_γ_h_) with an EC_50_ of ∼0.3 μM and by ∼2-fold at the near-saturating S3969 concentration of 10 μM. These observations are in good agreement with previous reports (43, 45). In vehicle- and time-matched control experiments, no spontaneous increase of ENaC currents occurred (Fig. S1). This indicates that the observed increase of ΔI_ami_ in the presence of S3969 is due to a specific stimulatory effect of the small molecule activator on the channel. In contrast to human ENaC, mouse ENaC (α_m_β_m_γ_m_) was insensitive to S3969 in concentrations up to 10 μM (Fig. 1*B*). To demonstrate that this lack of effect of S3969 was not due to an exceedingly high baseline open probability of the murine channel, we used chymotrypsin as an alternative tool to increase ENaC open probability by proteolytically cleaving the channel’s γ-subunit (11, 12, 13, 14). As previously reported (46), chymotrypsin stimulated ΔI_ami_ in α_m_β_m_γ_m_-expressing oocytes in a similar manner as in α_h_β_h_γ_h_-expressing oocytes (Fig. S2). This indicates that baseline open probability of both murine and human ENaC is significantly lower than 1. Thus, the lack of effect of S3969 on murine ENaC cannot be explained by a constitutively high open probability of this channel.

Importantly, coexpression of mouse α- and γ-ENaC with human β-ENaC (α_m_β_h_γ_m_; Fig. 1*C*) or with a chimeric mouse β-ENaC subunit, in which the ECL was replaced with that of human β-ENaC (residues 72–515; α_m_β_m,h(72−515)_γ_m_; Fig. 1*D*), resulted in channels which could be stimulated by S3969 with an estimated EC_50_ of ∼0.5 μM similar to that measured for α_h_β_h_γ_h_-ENaC. Taken together, our findings confirm previously published data that S3969 can stimulate human but not murine ENaC and that the critical S3969-binding site mediating this stimulatory effect is localized in the ECL of human β-ENaC.

### The proximal part of the thumb domain of β-ENaC is critically involved in channel stimulation by S3969

**Figure 2:**
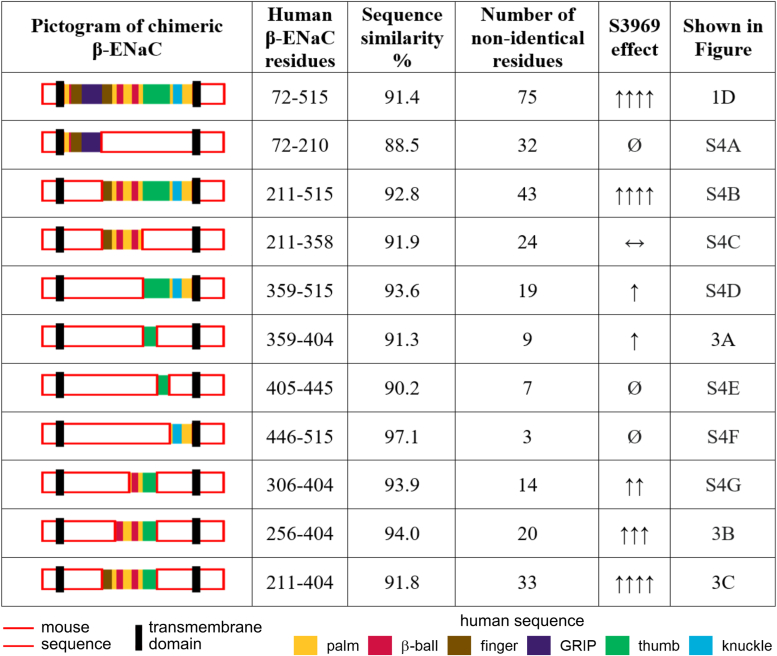
**Mouse-human chimeric β-ENaCs used to identify a portion of the ECL critically involved in the stimulatory effect of S3969.** The pictograms highlight in color the parts of the ECL in mouse β-ENaC that were replaced by the corresponding human ECL sequence. The different colors represent the domain organization with palm in *yellow*, β-ball in *red*, finger in *brown*, GRIP in *violet*, thumb in *green*, and knuckle in *blue*. Unmodified portions of mouse β-ENaC are shown in *white* with a *red* outline. Transmembrane domains are represented as *black* bars. Similarity between mouse and human sequences, as well as the number of nonidentical residues in the replaced channel portion, were calculated from the sequence alignment shown in Fig. S3. Ø: no stimulatory effect of S3969; ↔, marginal stimulatory effect with 10 μM of S3969; ↑, ↑↑, ↑↑↑, ↑↑↑↑: significant stimulatory effect of S3969 ranked according to the estimated EC_50_ values and/or maximal normalized ΔI_ami_ achieved with 10 μM of S3969. ENaC, epithelial sodium channel.

**Figure 3:**
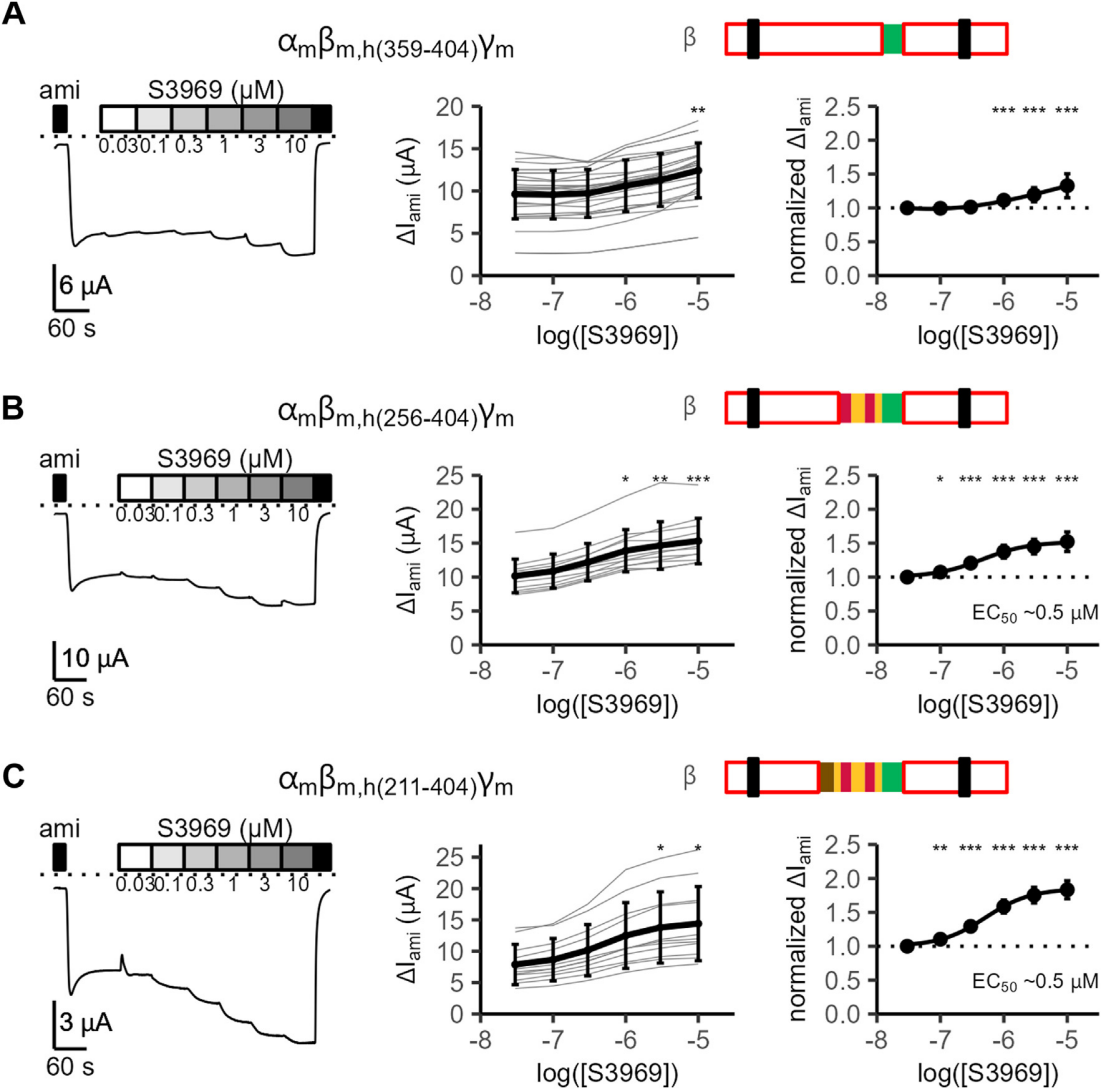
**Identification of a β-ECL portion critically involved in the stimulatory effect of S3969.***A*–*C*, *left panels*: representative whole-cell current traces obtained in individual oocytes expressing mouse α_m_- and γ_m_-subunits together with a chimeric mouse-human β subunit (β_m,h_) as indicated. Pictograms illustrate which portion of mouse β-ENaC were replaced by the corresponding region of human β-ENaC. The explanation for the color code is given in Figure 2. The experimental protocol was similar to that described in Figure 1. *Middle* and *right panels*: Concentration-response relationship of the stimulatory effect of S3969 on absolute (*middle panel*) or normalized ΔI_ami_ (*right panel*). Summary data obtained from similar experiments as shown in *left panels* and analyzed as described in Figure 1. Mean ± SD are shown. In *right panels*, mean values are fitted to a spline function (*A*: N = 5, n = 31; N indicates the number of different batches of oocytes, n indicates the number of individual oocytes studied per experimental group) or to Equation 1 (*B*: N = 2, n = 12; *C*: N = 2, n = 11). One-way ANOVA with Bonferroni’s post hoc test was used to calculate *p*-values for comparisons with the baseline current values: in *middle panels* (*A*: 1/1/1/1/0.0534/0.005; B: 1/1/1/0.021/0.002/0.0003; *C*: 1/1/1/0.265/0.040/0.015); in *right panels* (*A*: 1/1/1/<0.0001/<0.0001/<0.0001; *B*: 1/0.041/<0.0001/<0.0001/<0.0001/<0.0001; *C*: 1/0.002/<0.0001/<0.0001/<0.0001/<0.0001). ∗*p* < 0.05, ∗∗*p* < 0.01, and ∗∗∗*p* < 0.001 indicate significant stimulation by S3969. ENaC, epithelial sodium channel.

To narrow down the channel region responsible for the stimulatory effect of S3969, we generated a series of chimeric β-subunits by transplanting different portions of the extracellular loop of human β-ENaC into the corresponding regions of mouse β-ENaC (Fig. 2). Subsequently, each mouse-human chimeric β-ENaC was functionally tested in co-expression experiments with WT mouse α- and γ-subunits. The effect of S3969 on these channels was investigated using a similar protocol as described in Figure 1. First, we tested whether the transplantation of the proximal portion of human β-ECL containing the GRIP domain and a part of the finger domain (residues 72–210; α_m_β_m,h(72−210)_γ_m_) could convert mouse ENaC into an S3969-sensitive channel. This transplanted portion includes the GRIP domain and contains 32 of 75 amino acid residues that differ in the ECL of human *versus* mouse βENaC (Figs. S3 and 2). The chimeric α_m_β_m,h(72−210)_γ_m_-ENaC was functional but could not be stimulated by S3969 at concentrations up to 10 μM (Fig. S4*A*). In contrast, a chimeric channel containing the middle and the distal portion of human β-ECL (residues 211–515; α_m_β_m,h(211−515)_γ_m_) could be strongly stimulated by S3969 with an estimated EC_50_ value similar to that observed in WT human ENaC (∼0.5 μM; Fig. S4*B*). These findings suggest that residues 211 to 515 of the ECL of human β-ENaC include the residues critical for mediating the stimulatory effect of S3969. Thus, this portion was further subdivided approximately into two halves corresponding to the middle and the distal part of the ECL (residues 211–358 and 359–515, respectively). Interestingly, S3969 only had a marginal effect on α_m_β_m,h(211−358)_γ_m_-ENaC even when applied in a concentration of 10 μM (Fig. S4*C*). In contrast, the other chimeric channel containing the distal part of the ECL (α_m_β_m,h(359−515)_γ_m_) could be substantially stimulated by S3969. However, the apparent affinity of S3969 to this channel appeared to be reduced in comparison with WT human ENaC, as a saturating concentration was not reached with 10 μM of S3969 (Fig. S4*D*). Nevertheless, this suggests that residues 359 to 515 of the human β-ENaC ECL include functionally important residues for mediating the stimulatory effect of S3969. Investigation of additional chimeric channels revealed that it was sufficient to introduce the first third of this human sequence into mouse β-ENaC (*i.e.*, residues 359–404; α_m_β_m,h(359−404)_γ_m_; Fig. 3*A*) to achieve a similar partial stimulatory effect of S3969. This sequence forms a proximal part of the β-ENaC thumb domain. In contrast, transplantation of residues 405 to 445 or 446 to 515 of human β-ENaC, corresponding to the distal part of the thumb domain or the knuckle and part of the palm domain, respectively, did not result in the formation of S3969-sensitive channels (Fig. S4, *E* and *F*). Thus, we can conclude that residues 359 to 404 in the ECL of human β-ENaC, forming the proximal part of the channel's thumb domain, are critical for the stimulatory effect of S3969 on ENaC.

However, transplanting this part of human β-ENaC (residues 359–404) into mouse β-ENaC did not convey full responsiveness to S3969 (Fig. 3*A*). Taking into account that the middle portion of the β-ECL (residues 211–358) also contributed, at least in part, to the stimulatory effect of S3969 (Fig. S4*C*), we sequentially extended the transplanted human sequence to replace not only the proximal part of the thumb domain but also parts of the palm, β-ball, and finger domains in mouse β-ENaC (Fig. 2, *bottom three rows*). This successively increased ENaC affinity to S3969 (Figs. S4*G* and 3, *B* and *C*). With α_m_β_m,h(256−404)_γ_m_-ENaC, an EC_50_ value of ∼0.5 μM was eventually reached (Fig. 3*B*) similar to that observed in channels containing WT human β-ENaC (Fig. 1, *A* and *C*) or its entire ECL (Fig. 1*D*). A further extension of the transplanted region (α_m_β_m,h(211−404)_γ_m_-ENaC, Fig. 3*C*) had no additional effect on ENaC affinity to S3969 (EC_50_ ∼0.5 μM) but slightly enhanced the relative stimulatory effect of S3969 possibly due to a lower baseline open probability of this chimeric channel. It is noteworthy that the two chimeric channels shown in Figure 3, *A* and *B* have similar baseline currents but different apparent affinities to S3969. Thus, the different responsiveness of these ENaC chimeras to S3969 cannot be attributed to differences in baseline currents.

Overall, these data indicate that the proximal part of the thumb domain of human β-ENaC is critically involved in channel stimulation by S3969. In addition, portions of the palm, β-ball, and finger domains also contribute to mediating ENaC stimulation by S3969.

### The arginine residue (R388) in the α4 helix of the thumb domain of human β-ENaC is important for the stimulatory effect of S3969

**Figure 4:**
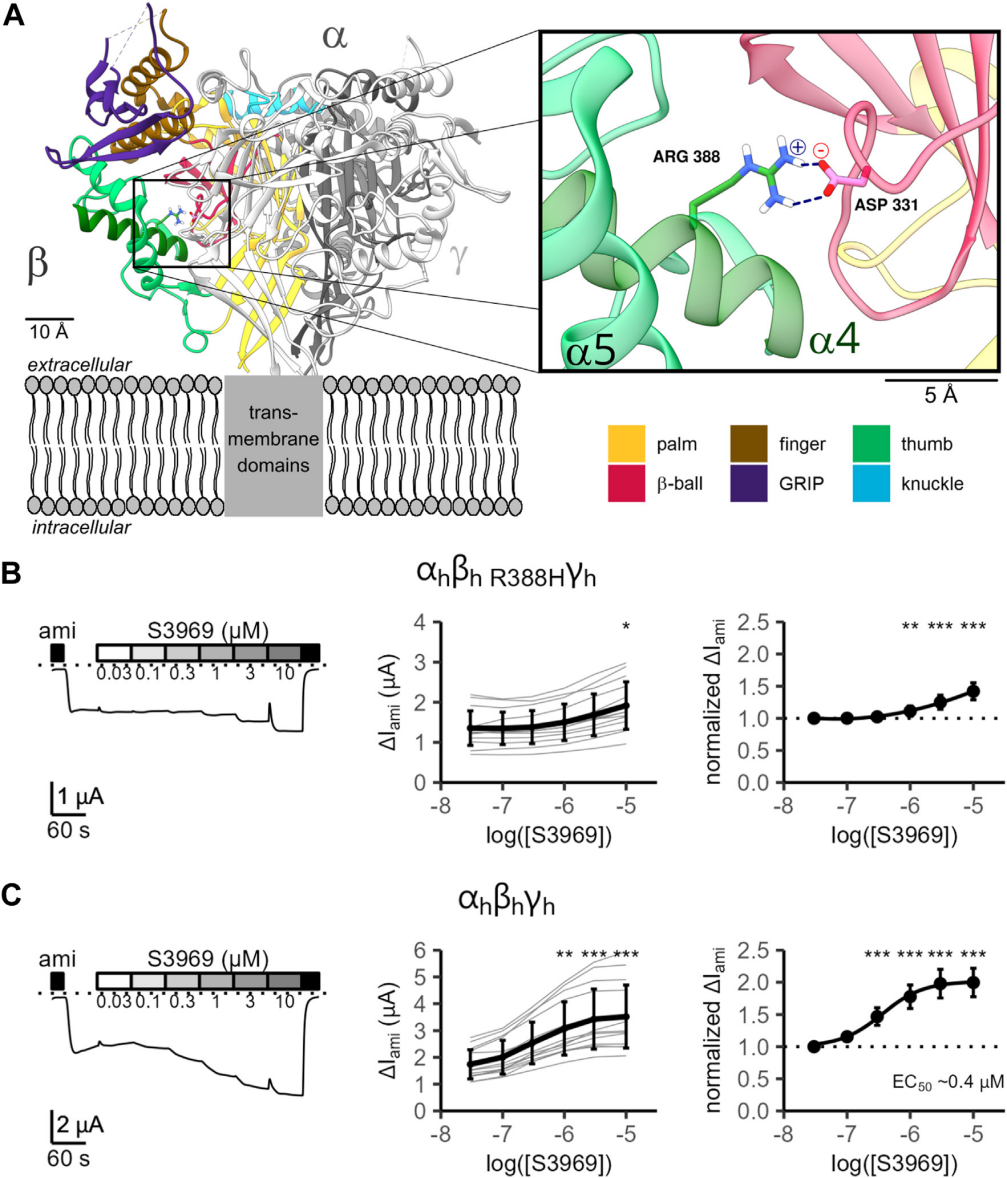
**An arginine residue (Arg388) in β-ENaC is functionally important for channel stimulation by S3969.***A*, ribbon diagram of the ECL of human αβγ-ENaC generated using atom coordinates from PDB entry 6WTH (8, 71). α-subunit is shown in *dark gray*, γ-subunit in *white*, and β-subunit is colored according to its domain organization as indicated. The putative location of unresolved transmembrane domains is indicated with a *gray box* placed within a schematically depicted lipid bilayer. The inset shows the location of a salt bridge interaction (visualized by *blue dotted* lines and electric charge symbols) between an arginine residue Arg388 in the α4 helix of the thumb domain and an aspartate residue Asp331 in the β-ball domain. Both residues are shown in stick representation with carbon atoms in *green* for Arg388 or *pink* for Asp331, nitrogen in *blue*, oxygen in *red*, and hydrogen in *white*. Nonpolar hydrogen atoms are omitted for clarity. *B* and *C*, representative whole-cell current trace (*left panel*) obtained in an oocyte expressing the mutant human ENaC (α_h_β_h__R388H_γ_h_, *B*) or WT human ENaC (α_h_β_h_γ_h_, *C*) and summary data obtained from similar experiments showing the concentration-response relationship of the stimulatory effect of S3969 on absolute (*middle panel*) or normalized ΔI_ami_ (*right panel*). Analysis was performed as described in Figure 1. Mean ± SD are shown. In *right panels*, mean values are fitted to a spline function (*B*: N = 2, n = 15; N indicates the number of different batches of oocytes, n indicates the number of individual oocytes studied per experimental group) or to Equation 1 (*C*: N = 2, n = 15). One-way ANOVA with Bonferroni’s post hoc test was used to calculate *p*-values for comparisons with the baseline current values: in *middle panels* (*B*: 1/1/1/1/1/0.042; *C*: 1/1/0.0581/0.003/<0.0001/<0.0001); in *right panels* (*B*: 1/1/1/0.0096/<0.0001/<0.0001; *C*: 1/1/<0.0001/<0.0001/<0.0001/<0.0001). ∗*p* < 0.05, ∗∗*p* < 0.01, and ∗∗∗*p* < 0.001 indicate significant stimulation by S3969. ENaC, epithelial sodium channel.

Replacing the proximal part of the thumb domain in mouse β-ENaC by the corresponding portion of human β-ENaC (residues 359–404) was equivalent to the conversion of nine amino acid residues from the murine to human sequence (Figs. 2 and S3). Interestingly, analysis of the published structure of human ENaC revealed, that one of these residues, the arginine residue 388 (Arg388) localized in the α4 helix of the thumb domain, mediated an interdomain interaction between the thumb and the β-ball domain of β-ENaC by forming a salt bridge with the aspartate residue 331 (Asp331) (Fig. 4*A*). In mouse β-ENaC, the corresponding positions are occupied by a histidine (His386) and a glutamate (Glu329) residue, which probably form less stable interactions at physiological pH due to a low protonation state of the imidazole side chain of the histidine. We hypothesized that this salt bridge interaction in human β-ENaC stabilizes the local protein conformation at the thumb/β-ball domain interface, which may be necessary for S3969 binding. To test this, we substituted Arg388 with a histidine (R388H) or an alanine (R388A) residue in human β-ENaC and investigated whether these modifications weakened the S3969 effect. Indeed, both α_h_β_h R388H_γ_h_-ENaC (Fig. 4*B*) and α_h_β_h R388A_γ_h_-ENaC (Fig. S5) demonstrated a reduced S3969-mediated stimulation in comparison with WT human ENaC studied in parallel control experiments (Fig. 4*C*). Conversely, replacing the histidine residue (His386) in mouse β-ENaC with an arginine conferred partial S3969 sensitivity to the mouse channel (Fig. S6), which is consistent with the results obtained from the α_m_β_m,h(359−404)_γ_m_ chimeric channel (Fig. 3*A*).

Taken together, these data indicate that the arginine residue (Arg388) in the α4 helix of the thumb domain of human β-ENaC is important for channel stimulation by S3969. Moreover, combined with the results of our chimeric approach, they strongly suggest that a putative S3969-binding site is localized in the vicinity of the thumb/β-ball domain interface in the channel's β-subunit.

### Identification of a binding site for S3969 in β-ENaC

Further analysis of the β-ENaC structure in a surface representation revealed the presence of a cavity at the thumb/β-ball domain interface that could serve as a binding pocket for S3969 (Fig. S7*A*). It comprises an extensive lower area formed by the thumb and the β-ball domain and extends upwards as a narrower cleft reaching to the finger and palm domain. Interestingly, molecular docking predicted that an S3969 molecule would fit into both the upper and the lower part of this cavity (Fig. S7, *B* and *C*). It is noteworthy that the best binding mode in the lower region was ranked significantly higher (docking score: −8.2 kcal/mol; Fig. S7*C*) than that in the upper region (docking score: −3.8 kcal/mol; Fig. S7*B*), suggesting that the former represents a more energetically favorable S3969 interaction with the channel. Thus, for further analysis, we focused on the putative S3969-binding site in the lower part of the cavity.

**Figure 5:**
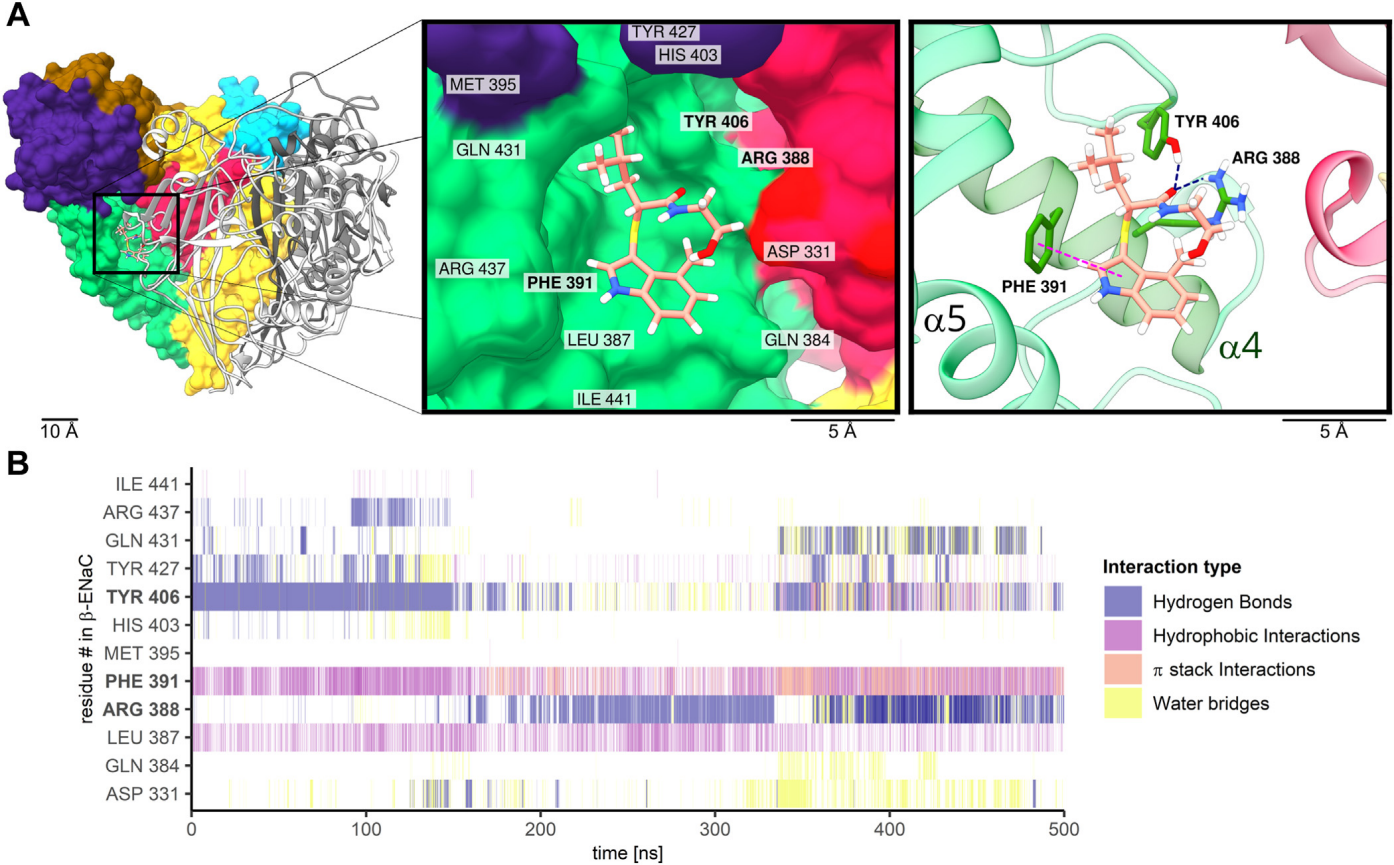
**Atomistic molecular dynamics simulations reveal stable interactions between S3969 and Arg388, Phe391 and Tyr406 residues in β-ENaC.***A*, putative binding site of S3969 in the lower part of the cavity localized in the ECL of human β-ENaC. Snapshot is taken from the same MD simulation as in (*B*) at *t* = 405 ns. β-subunit is shown in surface representation and colored according to its domain organization using the same color code as in Figure 2. α- and γ-subunits are in ribbon representation in *dark gray* and *white*, respectively. S3969 is shown in *stick* representation with carbon atoms in *tan*, nitrogen in *blue*, oxygen in *red*, sulfur in *yellow*, and hydrogen in *white*. Insets show the S3969-binding site on an expanded scale in surface (*left inset*) or ribbon representation (*right inset*). In the *right inset*, Arg388, Phe391, and Tyr406 residues forming the most stable interactions with S3969 are shown in stick representation with carbon atoms in *green*, oxygen in *red*, nitrogen in *blue*, and polar hydrogen in *white*. Apolar hydrogen atoms of β-ENaC are omitted for clarity. *Blue* and *pink dotted lines* visualize hydrogen bond and hydrophobic interactions, respectively. *B*, diagram shows interactions formed between β-ENaC residues, which contribute to the putative S3969-binding site and labeled in the *left inset* in (*A*), and a S3969 molecule during 500 ns MD simulations. Interaction types are represented by different colors as indicated. Each line represents the formation of at least one interaction of the specified type per trajectory frame. A darker color intensity indicates multiple interactions of the same type per trajectory frame. ENaC, epithelial sodium channel.

**Figure 6:**
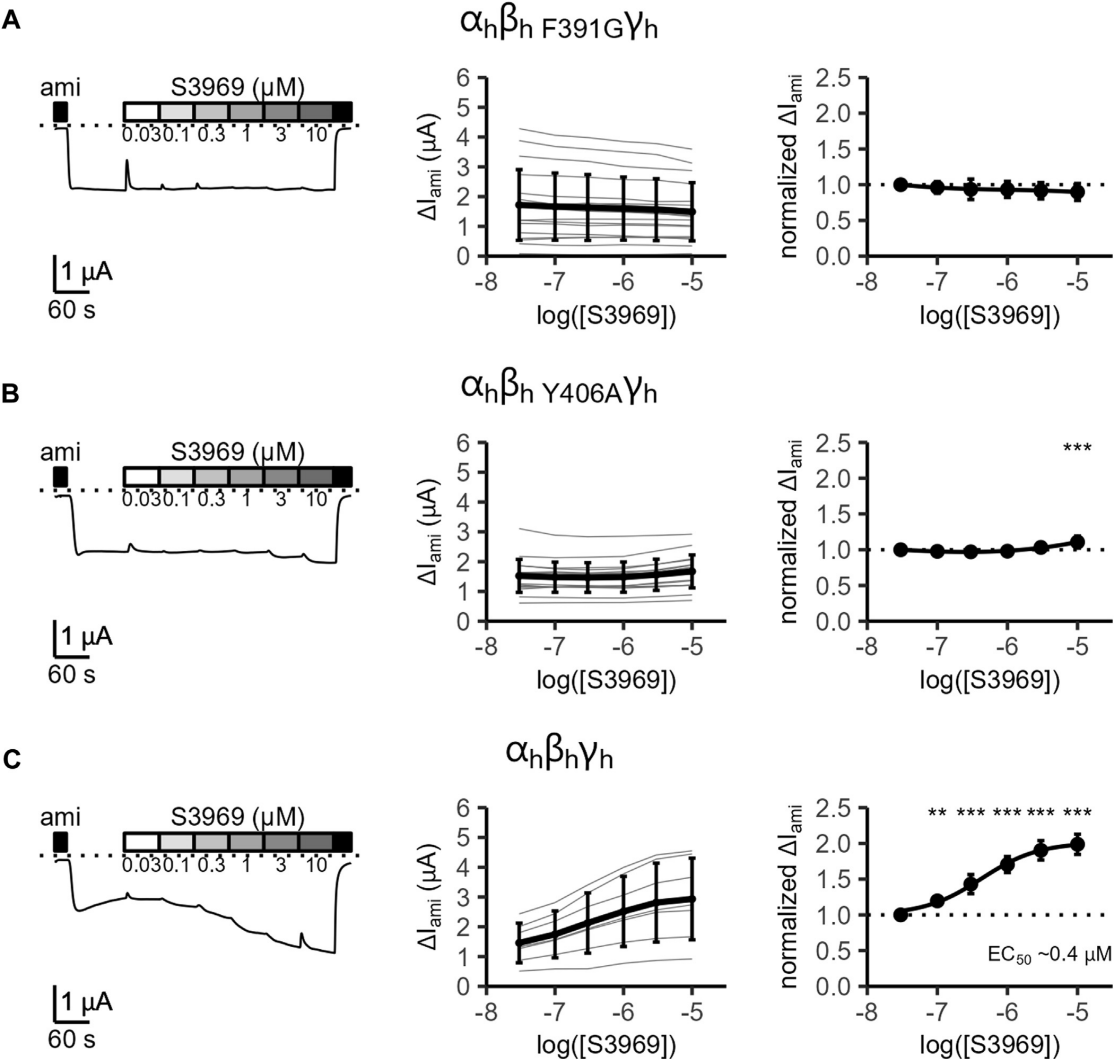
**Mutations F391G or Y406A in human β-ENaC nearly abolish the stimulatory effect of S3969.***A*–*C*, representative whole-cell current traces (*left panels*) obtained in individual oocytes expressing mutant human ENaC (α_h_β_h__F391G_γ_h_ in *A* or α_h_β_h__Y406A_γ_h_ in *B*) or WT human ENaC (α_h_β_h_γ_h_, *C*) and summary data obtained from similar experiments showing concentration-response relationships of the stimulatory effect of S3969 on absolute (*middle panel*) or normalized ΔI_ami_ (*right panel*). Analysis was performed as described in Figure 1. Mean ± SD are shown. In *right panels*, mean values are fitted to a spline function (*A*: N = 3, n = 18; *B*: N = 3, n = 18; N indicates the number of different batches of oocytes, n indicates the number of individual oocytes studied per experimental group) or to Equation 1 (*C*: N = 3, n = 7). One-way ANOVA with Bonferroni’s post hoc test was used to calculate *p*-values for comparisons with the baseline current values: in *middle panels* (*A*: 1/1/1/1/1/1; *B*: 1/1/1/1/1/1; *C*: 1/1/1/1/0.364/0.212); in *right panels* (*A*: 1/1/1/1/1/1; *B*: 1/1/0.725/1/1/<0.0001; *C*: 1/0.006/<0.0001/<0.0001/<0.0001/<0.0001). ∗∗*p* < 0.01 and ∗∗∗*p* < 0.001 indicate significant stimulation by S3969. ENaC, epithelial sodium channel.

To assess the stability of this docked S3969-ENaC complex and to identify residues coordinating S3969, we performed atomistic MD simulations. Importantly, during the 500 ns simulation time, S3969 remained bound to the binding pocket (Fig. 5*A*) and was stabilized by forming flexible hydrogen bond interactions between its carbonyl-oxygen and a tyrosine (Tyr406) and/or the aforementioned arginine (Arg388) residue. Thus, the computer predictions were in good concordance with the experimental findings shown in Figure 4*B*. In addition, we observed stable hydrophobic and π-stack interactions between the indole-group of S3969 and a phenylalanine residue (Phe391; Fig. 5*B*). Importantly, like Arg388, Phe391 is also localized in the α4 helix of the thumb domain. To validate these MD predictions in functional experiments, Tyr406 and Phe391 were mutated individually, and the effect of S3969 was investigated in oocytes expressing the corresponding mutant channels. Phe391 was substituted with a glycine residue, which lacks the side chain and therefore is unable to form hydrophobic and π-stack interactions with S3969. Tyr406 was replaced with an apolar alanine residue to prevent hydrogen bond interactions with S3969. Importantly, the former mutation (β_h F391G_; Fig. 6*A*) almost abolished, and the latter mutation (β_h Y406A_; Fig. 6*B*) largely reduced the S3969-mediated ENaC activation compared to that observed in control experiments with WT human ENaC (Fig. 6*C*). It is noteworthy that the stimulatory effect of chymotrypsin was preserved in channels with mutations (β_h R388A_; β_h F391G_; β_h Y406A_) that significantly reduced the responsiveness to S3969 (Fig. S8). This indicates that the reduced stimulatory effect of S3969 is not due to an increased open probability but that the mutations specifically hinder a stimulatory interaction of S3969 with the mutant channels. Taken together, these findings indicate that the thumb domain residues Arg388, Phe391, and Tyr406 in human β-ENaC are critically involved in mediating channel stimulation by S3969.

As an additional control, we performed MD simulations of the other docked S3969–ENaC complex, in which S3969 was localized in the upper cleft (Fig. S7*B*). Despite a significantly lower docking score, S3969 remained stably bound to ENaC due to adjustments of the ENaC structure to accommodate S3969, which occurred during the first ∼50 ns of the simulation time (Fig. S9*A*). S3969 was coordinated mainly by an arginine (Arg330) and a tyrosine (Tyr427) residue *via* hydrogen bonds and water bridges (Fig. S9*B*). However, mutating these residues resulted only in a marginal reduction of the S3969 affinity (Fig. S10), arguing against a critical role of the upper cleft as a functionally relevant S3969-binding site in human β-ENaC.

Taken together, we have identified the likely stimulatory binding site for S3969 in the β-subunit of human ENaC. In this binding site, the α4 helix of the thumb domain seems to play a critical role in coordinating the S3969 molecule.

### A putative molecular mechanism involved in ENaC stimulation by S3969

**Figure 7:**
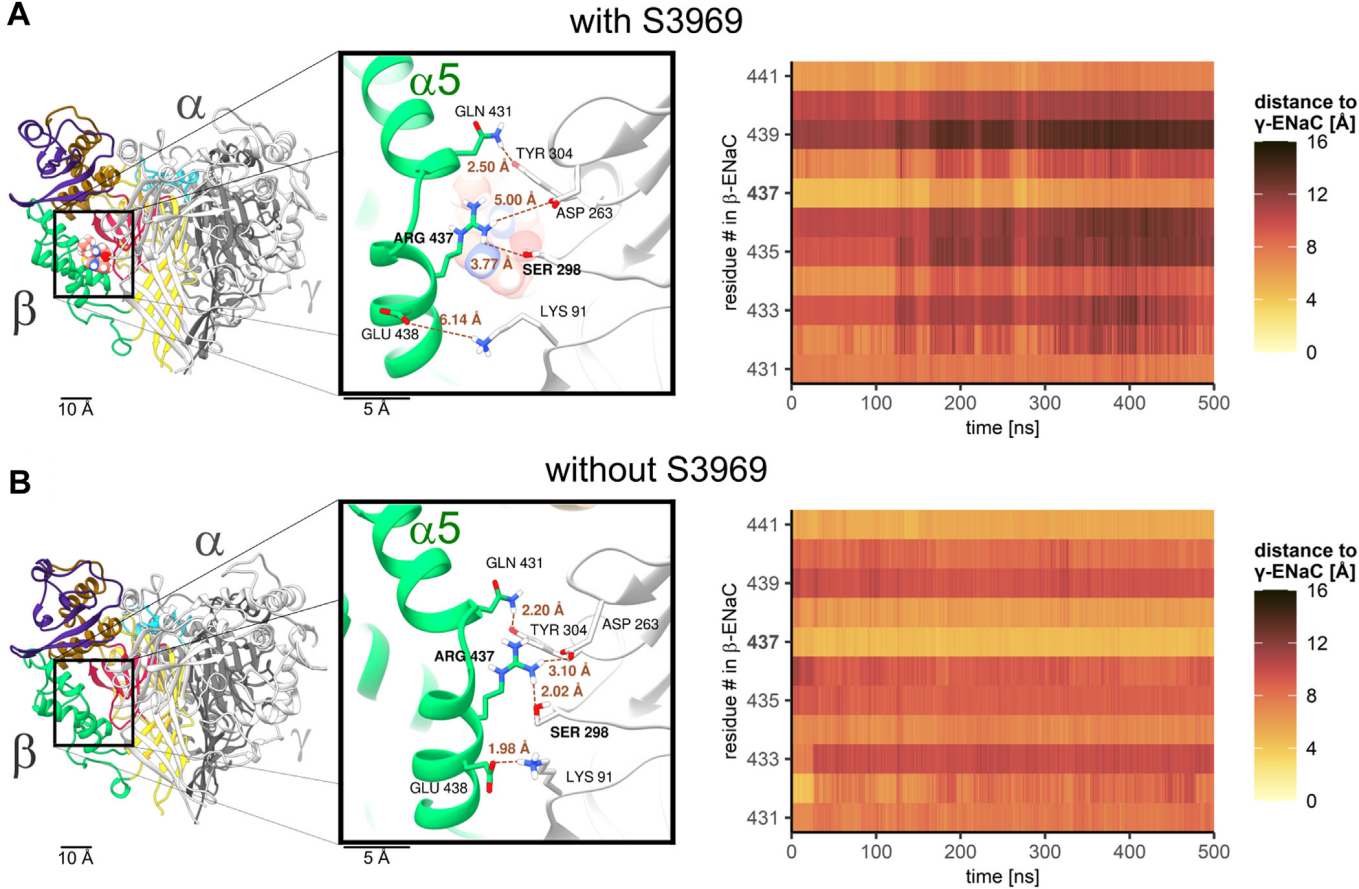
**Binding of S3969 increases the distance between the α5 helix of the thumb domain in β-ENaC and the palm domain in γ-ENaC.***A* and *B*, *left panels*: Human αβγ-ENaC was simulated with (*A*) or without (*B*) S3969. Snapshots are taken from corresponding MD simulations at *t* = 405 ns. ENaC is shown in ribbon representation and S3969 in spheres representation using the same color code as in Figure 2. The insets show a part of the β-γ-subunit interface at a larger scale to illustrate the conformational change of the α5 helix of the thumb domain of β-ENaC (in *green*), elicited by S3969 binding. Side chains of selected residues are shown in stick representation to illustrate the increase in the intersubunit distance caused by S3969. Distances were calculated between the selected atoms as indicated by *dotted brown* lines. Carbon atoms of β-ENaC are in *green*, of γ-ENaC in *white*, nitrogen atoms in *blue*, oxygen in *red*, and hydrogen in *white*. Apolar hydrogen atoms are omitted for clarity. *Right panels*: For each β-ENaC residue from 431 to 441, the distance between its center of mass and the center of mass of the closest residue in γ-ENaC was calculated in each MD trajectory frame. The distance values are represented by different colors using the scientific color map “lajolla” (76) shown on the *right*. ENaC, epithelial sodium channel.

The binding of S3969 to the thumb domain of β-ENaC is expected to cause a local conformational change, which is probably propagated towards the transmembrane domains, ultimately leading to channel opening and/or hindering channel closing. To address the question which local conformational changes are triggered by S3969 binding, we re-evaluated our MD simulations of ENaC in complex with S3969 described above. We compared the results with those obtained in control simulations of ENaC in the absence of S3969. We observed no substantial conformational changes of the S3969-binding site during 500 ns MD simulations. In particular, the salt bridge interaction between Arg388 and Asp331, that connected the thumb- and β-ball domains of β-ENaC, remained stable. Interestingly, we observed a substantial movement of the α5 helix of the thumb domain away from the neighboring γ-subunit. This shift led to a progressive increase in distance between thumb domain residues 431 to 441 of β-ENaC and adjacent palm domain residues of γ-ENaC (Fig. 7*A*). In control simulations performed without S3969, no such movement of the α5 helix was observed (Fig. 7*B*). Thus, binding of S3969 appears to enlarge the β-γ-intersubunit distance at this site.

**Figure 8:**
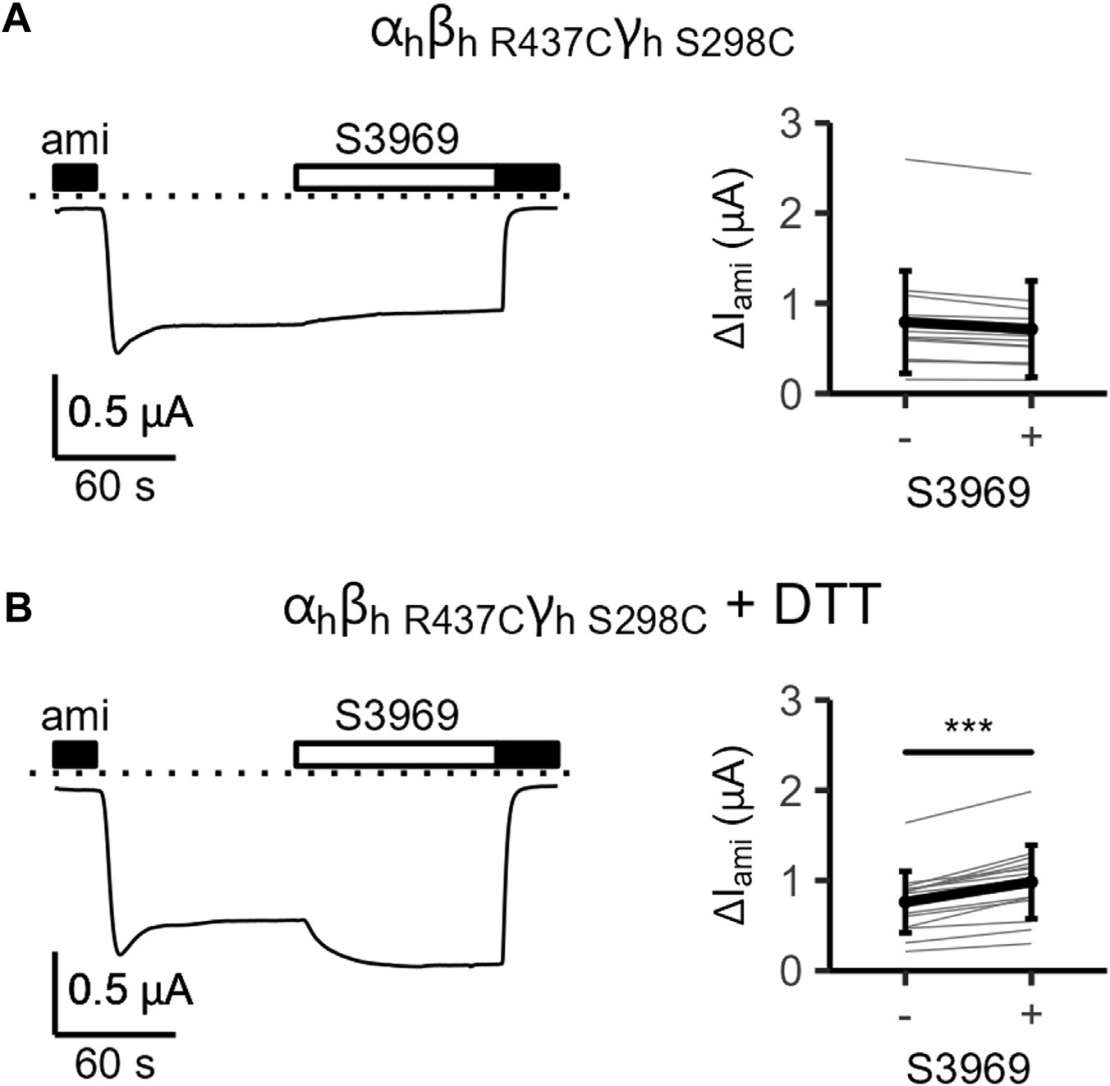
**Replacing Arg437 in β- and Ser298 in γ-ENaC with cysteines to form a disulfide-bond prevents ENaC stimulation by S3969 which can be rescued by DTT.***A* and *B*, *left panel*: Representative whole-cell current traces are shown for an oocyte expressing mutant human ENaC (α_h_β_h__R437C_γ_h S298C_) without (*A*) or with (*B*) 15 min pre-incubation in 30 mM DTT. Amiloride (ami, 2 μM) and S3969 (10 μM) were present in the bath solution as indicated by *black* and *white* bars, respectively. *Dotted lines* indicate zero current level. *Right panel*: Summary of ΔI_ami_ values measured before (−) and after (+) application of S3969 from similar experiments as shown in the corresponding *left panel*. *Gray lines* connect data points obtained in an individual oocyte. Mean ± SD are shown in *black* (N = 2, n = 15; N indicates the number of different batches of oocytes, n indicates the number of individual oocytes studied per experimental group). Paired *t* test was used to calculate *p*-values (*A*: <0.0001, *B*: <0.0001). ∗∗∗*p* < 0.001 indicates significant stimulation by S3969. ENaC, epithelial sodium channel.

To confirm this prediction experimentally, we replaced Arg437 in the α5 helix of the thumb domain of human β-ENaC and Ser298 in the palm domain of human γ-ENaC with cysteines (β_h R437C_, γ_h__S298C_). Due to their proximity, these two cysteine residues are expected to form spontaneously a disulfide bond. We hypothesized that introducing this disulfide bond would prevent the S3969-induced increase of the β-γ-intersubunit distance and thereby compromise channel activation. Indeed, α_h_β_h R437C_γ_h S298C_-ENaC could not be activated by 10 μM S3969 (Fig. 8*A*). In contrast, mutant channels in which only a single cysteine residue was introduced either in β- (α_h_β_h R437C_γ_h_; Fig. S11*A*) or γ-ENaC (α_h_β_h_γ_h S298C_; Fig. S11*B*) remained sensitive to S3969. If accessible, newly introduced disulfide bonds in the ECL of ENaC can be reduced by incubating oocytes with DTT (14). Importantly, pre-incubation of oocytes with DTT (30 mM, 15 min) restored, at least in part, the stimulatory effect of S3969 in the double cysteine mutant channel (Fig. 8*B*). In additional control experiments, we demonstrated that pre-incubation of oocytes expressing the WT human ENaC with DTT did not significantly affect ENaC activation by S3969 (Fig. S11, *C* and *D*). This indicates that DTT specifically rescued the stimulatory response of α_h_β_h R437C_γ_h S298C_-ENaC to S3969 due to its reducing effect on the disulfide bond between Arg437 and Ser298. Removal of this covalent linkage by DTT probably restored the flexibility of the α5 helix of the thumb domain in β-ENaC, which appears to be necessary for ENaC stimulation by S3969.

Taken together, the results of computer simulations and functional experiments suggest that the movement of the α5 helix of the thumb domain of β-ENaC away from the palm domain of γ-ENaC is a putative molecular mechanism involved in S3969-mediated ENaC stimulation.

### S3969 stimulates ENaC-mediated electrogenic transport in H441 airway epithelial cells

**Figure 9:**
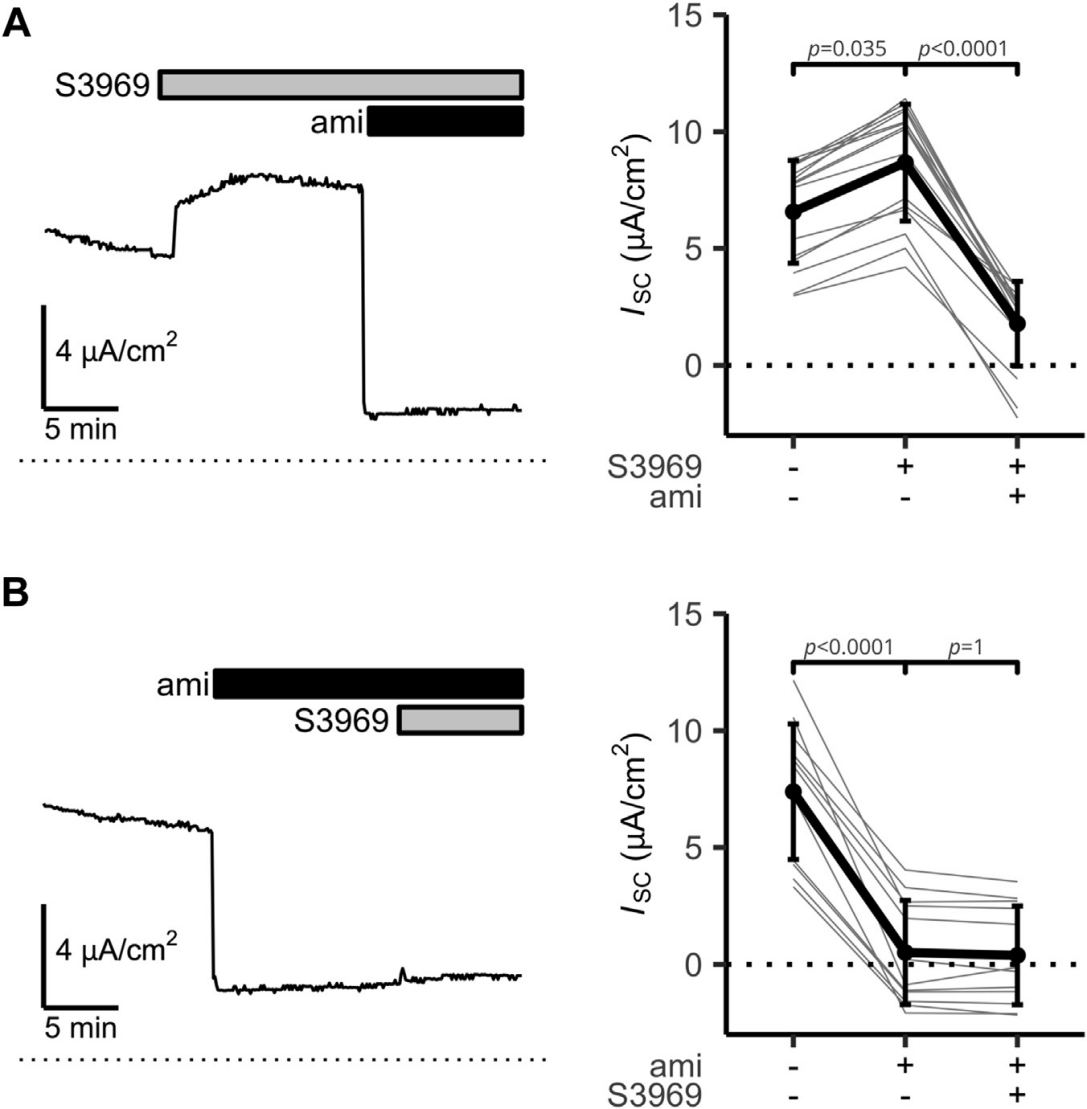
**S3969 stimulates ENaC in H441 cells.***A* and *B*, *left panel*: Representative equivalent short circuit current (*I*_SC_) recordings are shown. S3969 (10 μM) and amiloride (ami, 10 μM) were present in the apical bath solution as indicated by *gray* and *black bars*, respectively. Initial parts of recordings (∼30 min) corresponding to the equilibration phase after transferring the cells into Ussing chambers and applying Ringer's solution to the apical compartment are omitted for clarity. The *dotted lines* indicate zero current level. *Right panel*: Summary data obtained in similar experiments as shown in the *left panel* demonstrate the effect of S3969 on *I*_SC_ in the absence (*A*) or the presence (*B*) of apical amiloride. *I*_SC_ values were measured in each individual recording before the application of S3969 (*A*) or amiloride (*B*), before the subsequent application of amiloride in the presence of S3969 (*A*) or of S3969 in the presence of amiloride (*B*), and at the end of the experiment. *Gray lines* connect data points obtained from an individual H441 cell monolayer. Mean ± SD are shown in *black* (*A*: n = 14; *B*: n = 12). One-way ANOVA with Bonferroni’s post hoc test was used to calculate *p*-values. ENaC, epithelial sodium channel.

To test, whether S3969 can stimulate ENaC endogenously expressed in a human epithelial cell line, we performed proof-of-principle experiments using H441 human distal lung epithelial cells. To assess ENaC-mediated electrogenic transepithelial sodium transport, we performed equivalent short-circuit current (*I*_SC_) measurements in modified Ussing chambers. Figure 9*A* shows a representative *I*_SC_ recording and corresponding summary data obtained in similar experiments, in which 10 μM S3969 was applied to the apical compartment of H441 cells, followed by the apical application of 10 μM of amiloride. Notably, S3969 significantly increased baseline *I*_SC_ by 2.1 ± 0.6 μA/cm^2^. Subsequent application of amiloride caused an *I*_SC_ decrease averaging 7.1 ± 1.6 μA/cm^2^. Importantly, S3969 failed to stimulate *I*_SC_ in the presence of amiloride (Fig. 9*B*). This indicates that the *I*_SC_ component stimulated by apical S3969 in the absence of amiloride (Fig. 9*A*) was due to ENaC-mediated electrogenic transepithelial Na^+^ absorption. No major changes in transepithelial resistance were observed during these measurements (Fig. S12), which suggests that S3969 did not compromise the integrity of the cell monolayer. We conclude that endogenously expressed ENaC can be stimulated by S3969 in H441 airway epithelial cells by about 30 to 40%. This finding suggests that S3969 and/or related substances can affect ENaC activity in native epithelia *in vivo*.

## Discussion

This study combined structure-based simulations with molecular biological methods and electrophysiological measurements to investigate mechanisms involved in αβγ-ENaC stimulation by the small molecule activator S3969. First, we confirmed previously reported findings that S3969 activates human but not mouse αβγ-ENaC in low micromolar concentrations and that the stimulatory effect critically depends on the ECL of human β-ENaC (43). Next, we identified and characterized an S3969-binding site in human β-ENaC. Moreover, using MD simulations, we suggested a molecular mechanism for ENaC stimulation by S3969, which appears to involve a specific conformational change of the thumb domain of β-ENaC associated with an increased β-γ-intersubunit distance. Finally, we demonstrated that S3969 stimulated ENaC-mediated electrogenic transepithelial Na^+^-transport in H441 human airway epithelial cells. Overall, our study highlights the important role of the β-subunit in regulating ENaC function. Moreover, it suggests that the molecular mechanism underlying the stimulatory effect of S3969 is functionally operative in human epithelial cells endogenously expressing ENaC.

As a starting point, we aimed to reproduce some previously reported observations regarding the stimulatory effect of S3969 on ENaC (43, 45). Using the *X. laevis* oocyte expression system and whole-cell current recordings, we demonstrated that heterologously expressed human but not mouse αβγ-ENaC can be stimulated by S3969 in a concentration-dependent manner. The estimated EC_50_ of ∼0.3 μM in our experiments was of the same order of magnitude as previously reported values (∼1.2 μM (43); ∼0.9 μM (45)). In the current study, S3969 application at the saturating concentration of 10 μM resulted in a ∼2-fold stimulation of baseline ENaC currents. This degree of stimulation was lower than the ∼7-fold stimulatory effect reported before (43). Since S3969 probably activates ENaC by increasing channel open probability ($P_{O}$) close to one (43), the relative stimulatory effect of S3969 is likely to correlate negatively with baseline ENaC $P_{O}$. Thus, the different relative stimulatory effect of S3969 in the two studies may be explained by different baseline ENaC $P_{O}$ values due to subtle differences in the experimental conditions used. In particular, in the present study, oocytes were kept in a bath solution with a low Na^+^ concentration (ND9) after cRNA injection to minimize Na^+^ loading of the oocytes and consequently Na^+^ feedback inhibition. In contrast, Lu *et al.* (43) used an incubation solution containing 96 mM Na^+^ (ND96). Thus, it is quite likely that ENaC $P_{O}$ was lower in their study than in the present investigation (47). This may well explain the different relative size of the S3969 stimulatory effects observed. Importantly, the stimulatory effects were qualitatively similar. Moreover, we confirmed that replacing the ECL of mouse β-ENaC with that of human β-ENaC converted mouse ENaC into an S3969-sensitive channel (43). We also demonstrated that the apparent EC_50_ of this chimeric channel was similar to that of human ENaC. Taken together, these results were in good agreement with previous reports and suggested that the binding site for S3969 is localized in the ECL of human β-ENaC.

To narrow down the channel region responsible for the stimulatory effect of S3969, we generated and functionally tested a series of mouse-human chimeric channels, in which different portions of the ECL of human β-ENaC were transplanted into the corresponding regions of mouse β-ENaC. Finally, using structure-based docking followed by 500 ns scale MD simulations in combination with site-directed mutagenesis and electrophysiological analysis of ENaC mutants, we identified an S3969-binding site in human β-ENaC. It was partially formed by the α4 helix of the thumb domain and localized at the β-γ-subunit interface. The conformation of this binding site appeared to be stabilized by a salt–bridge interaction between the Arg388 residue in the α4 helix of the thumb domain and the Asp331 residue in the β-ball domain. It is conceivable that in mouse ENaC, the conformation of the corresponding region does not favor S3969 binding. This may be explained, at least in part, by a weaker interaction between the thumb and β-ball domains due to the absence of this arginine residue (Arg 388) in mouse β-ENaC. Interestingly, the putative S3969-binding site was localized in the lower part of a large cavity formed by portions of the thumb, β-ball, finger, and palm domains of human β-ENaC. Our experiments did not provide any evidence for an involvement of the upper part of this cavity in mediating the S3969 effect. Interestingly, in ASICs, which are also members of the ENaC/DEG ion channel family, the region corresponding to the upper part of this β-ENaC cavity is an acidic pocket which represents the essential pH sensor of the channel (9, 48, 49, 50, 51, 52). Moreover, it serves as a binding site for two allosteric ASIC antagonists (53). Therefore, it is tempting to speculate that both the upper and lower part of this cavity in β-ENaC may serve as binding sites for negative and/or positive endogenous ENaC modulators, which remain to be identified. In line with this concept, one of the critical residues for S3969-mediated ENaC stimulation (Arg388) has previously been shown to contribute to chloride-mediated ENaC inhibition (54).

Which kind of molecular compounds may affect ENaC *via* the identified S3969-binding site? S3969 is a peptidomimetic with an indole ring, also found in tryptophan residues, and a leucine-serine dipeptide-like fragment (43). Our MD simulations suggested that the indole group and the carbonyl-oxygen of S3969 were critical for stabilizing the activator within its binding site. Thus, it is tempting to speculate that in native epithelia, locally occurring small peptides containing a tryptophan residue may interact with the putative S3969-binding site and act as endogenous ENaC modulators. Indeed, the modulation of some ENaC/DEG ion channels by small peptides is a well-characterized regulatory mechanism (1). In particular, FaNaC of *Helix aspersa* can be activated (55), and several ASICs can be modulated by FMRFamide and related peptides (1, 56).

Further clues regarding the nature of substances that may interact with the S3969-binding site were provided by a recent study in which five novel ENaC-activating compounds were identified using a screening approach (45). Similar to S3969, they all activated human but not mouse αβγ-ENaC and required human β-ENaC. Therefore, these substances may all act *via* the S3969-binding site identified in the present study. Notably, like S3969, four of the substances contained an indole ring as a distinctive feature, whereas in one compound, a benzothiophene ring was found instead (45). We propose that the identified putative S3969-binding site may be used for structure-based screening approaches to search for novel endogenous or pharmacological ENaC modulators. Particularly, this search should focus on indole and benzothiophene ring derivatives. An alternative strategy to develop new highly potent ENaC activators may be an optimization of the S3969 structure to improve its binding and functional properties.

**Figure 10:**
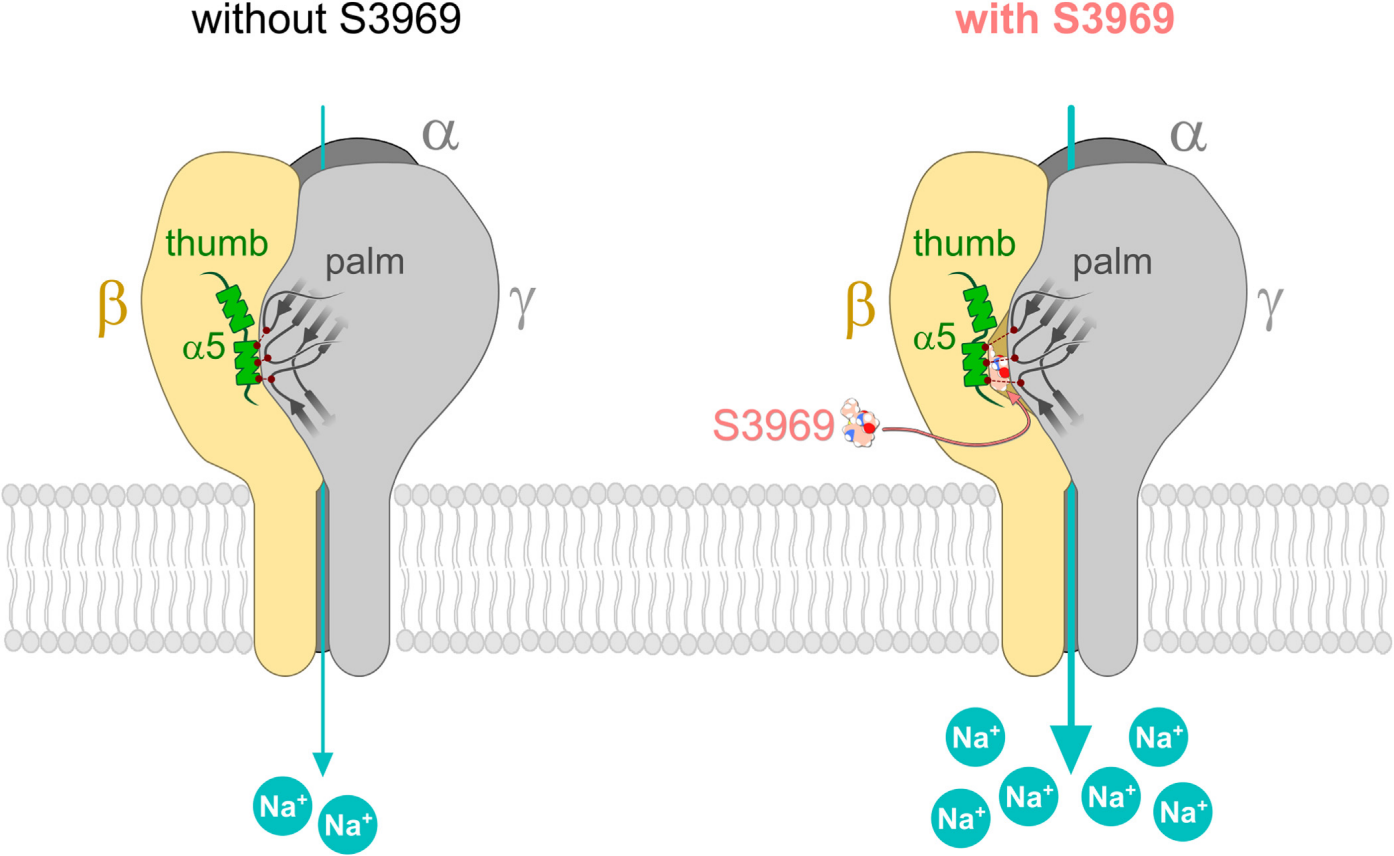
**Putative molecular mechanism underlying ENaC activation by S3969.** Binding of S3969 to β-ENaC moves the α5 helix of the thumb domain of β-ENaC away from the palm domain of γ-ENaC probably causing a conformational change resulting in channel activation. ENaC, epithelial sodium channel.

In addition to identifying the S3969-binding site, our study revealed a putative molecular mechanism involved in S3969-mediated ENaC stimulation. Our data suggest that the α5 helix of the thumb domain in β-ENaC moved away from the palm domain of γ-ENaC upon S3969 binding (Fig. 10). This is consistent with previous work by Collier *et al.*, (57) which indicated that conformational changes associated with an increased intersubunit distance favor the open state of ENaC. Interestingly, in the α- and γ-subunits, a loop connecting the α4 and α5 helix of the thumb domain is implicated in proteolytic channel activation by contributing to the binding site for the inhibitory GRIP domain fragments (7, 8, 14, 58). Thus, proteolytic ENaC activation may also be associated with structural rearrangements of the thumb domains albeit in α- and γ-ENaC. Interestingly, in ASICs, the α5 helix of the thumb domain contributes to the formation of the acidic pocket (9, 48, 49, 50, 51, 52). Moreover, it was demonstrated that the displacement of the α5 helix, leading to a collapse of the acidic pocket, induced channel activation of ASIC. Conversely, prevention of this collapse stabilized the closed state of the channel (48, 49, 50, 52, 53, 59). In contrast to our results in ENaC, the conformational change leading to ASIC opening was associated with a decrease in the intersubunit distance (48, 49, 50, 52, 53, 59). Therefore, ENaC and ASICs may have distinct gating mechanisms. Additional structural information on ENaC in different gating states is required to further confirm this hypothesis. It is also still unclear how the local conformational change of the thumb domain induced by S3969 is propagated to the transmembrane (TM) domains. This probably involves the so-called wrist region, which connects the ECL with the TM domains (7). The wrist region is thought to be critically involved in transmitting extracellular structural changes to the channel pore in both ASIC and ENaC (9, 60, 61, 62). However, the poor resolution of TM domains in the available ENaC structures is presently hindering a more detailed structural analysis of this issue (7, 8).

To date, the stimulatory effect of S3969 on ENaC currents has only been demonstrated in heterologous expression systems, like *Xenopus* oocytes, HEK293, and CHO cells (43, 44, 45, 63). Importantly, in the current work, we observed a significant stimulation of ENaC-mediated *I*_SC_ by S3969 in the confluent monolayers of human H441 respiratory epithelial cells endogenously expressing ENaC. Thus, S3969 can also affect human ENaC activity in differentiated and polarized epithelial cells.

In contrast to the stimulatory effect of S3969 observed in the present study, we previously found that under similar experimental conditions, apical application of exogenous proteases failed to stimulate ENaC in H441 cells (14, 64). This indicates that at the apical surface of these cells, proteolytic processing of ENaC is essentially complete under baseline conditions due to the activity of endogenous proteases. Thus, the stimulatory effect of S3969 in H441 cells appears to be additive to proteolytic ENaC stimulation. Its modest size (∼30–40%) indicates a relatively high baseline $P_{O}$ of ENaC in H441 cells consistent with substantial proteolytic pre-activation of the channel by endogenous proteases. Interestingly, in *Xenopus* oocytes, S3969 failed to stimulate ENaC after proteolytic channel activation by an exogenous protease (43). Therefore, our proof of principle experiments in H441 cells suggest that in epithelial cells with endogenous ENaC expression, unlike in oocytes heterologously expressing ENaC, S3969 and/or unknown S3969-like small molecules acting *via* the β-subunit may have a stimulatory effect on ENaC in addition to proteolytic channel activation. This additive effect of S3969 is conceptionally relevant with implications for the possible therapeutic usefulness of ENaC activators.

ENaC mediates transepithelial sodium absorption in lung epithelia, and the resulting osmotic gradient is critical for absorbing excess pulmonary fluid (17, 29, 65). Notably, we observed ENaC stimulation by S3969 in H441 cells under conditions of excess apical fluid. Therefore, pharmacological stimulation of ENaC may represent a promising therapeutic concept to improve pulmonary fluid clearance and ameliorate respiratory distress syndrome (32, 39, 41, 42). Patients suffering from severe hypotension and renal salt wasting due to PHA1B may also benefit from treatment with ENaC activators. Future studies are needed to evaluate the therapeutic potential of S3969 or similar compounds. Finally, diseases including certain forms of salt-sensitive hypertension or nephrotic syndrome are associated with inadequately high ENaC activity (18, 20, 66, 67, 68). In this context, it is tempting to speculate that ENaC stimulation *via* the S3969-binding site by not yet identified mediators may contribute to these pathophysiological states.

In conclusion, we have characterized the functional interaction of S3969 with ENaC at the molecular level. These findings may help to identify novel endogenous or pharmacological ENaC activators with potential pathophysiological relevance or therapeutic implications, respectively.

## Experimental procedures

### Chemicals

S3969 was synthesized essentially as described previously (43, 44). Bath solutions containing S3969 at a concentration ranging from 0.03 to 10 μM were prepared from 100 mM S3969 stock solution in DMSO on the day of the experiment. Amiloride and DTT were purchased from Sigma-Aldrich and were directly dissolved in the respective bath solution to the final concentration immediately before use.

### Isolation of oocytes and two-electrode voltage-clamp experiments

Full-length complementary DNAs (cDNAs) encoding human α-, β-, and γ-ENaC were kindly provided by Harry Cuppens. Full-length cDNAs for murine α-, β-, and γ-ENaC were kindly provided by Marcus A. Mall. cDNAs were subcloned into the pTLN vector for heterologous expression in *X. laevis* oocytes (69). Plasmids were linearized using MluI restriction enzymes (MluI-HF, New England Biolabs) and used as templates for cRNA synthesis using SP6 RNA polymerase (mMessage mMachine, Ambion). Mouse-human chimeric β-ENaCs were generated using NEBuilder HiFi DNA Assembly Master Mix (New England Biolabs). QuikChange Lightning site-directed mutagenesis kit (Agilent Technologies) was used to introduce point mutations. Sequences were routinely confirmed by sequence analysis (LGC Genomics).

Isolation of oocytes was essentially performed as described previously (14, 70). Ovarian lobes were excised by partial ovariectomy under anesthesia with Tricain 0.2%, in accordance with the principles of German legislation, with approval by the animal welfare officer for the University of Erlangen-Nürnberg (FAU), and under the governance of the state veterinary health inspectorate. Oocytes were isolated from ovarian lobes using a type-2 collagenase from *Clostridium histolyticum* (Sigma-Aldrich). Defolliculated stage V-VI oocytes were injected with 0.1 ng of cRNA per ENaC subunit (α, β, and γ) per oocyte. After cRNA injection, oocytes were kept in a low sodium ND9 solution (composition in millimolar: 9 NaCl, 2 KCl, 87 N-methyl-D-glutamine-Cl, 1.8 CaCl_2_, 1 MgCl_2_, 5 Hepes, and pH 7.4 adjusted with Tris) supplemented with 100 units/ml sodium penicillin and 100 μg/ml streptomycin sulfate. Two-electrode voltage-clamp measurements were performed 48 h after cRNA injection essentially as described previously (14, 70). Bath solution exchanges with a gravity-fed system were controlled by a magnetic valve system (ALA BPS-8; ALA Scientific Instruments) in combination with a TIB14 interface (HEKA). An individual oocyte was placed in an experimental chamber with a narrow flow channel (length: 45 mm; height: 3 mm; and width: 3 mm) with a U-shaped cross-section of ∼8 mm^2^. The oocyte was positioned in the experimental chamber close to the site of solution inflow and was held in place by the impaling microelectrodes. To achieve rapid and reproducible solution exchanges at the oocyte, the perfusion rate was carefully adjusted to ∼10 ml/min for each experimental solution, resulting in a flow velocity of ∼20 mm/s. The flow channel drained into a reservoir (2 cm × 1 cm) from which the solution was continuously removed *via* a suction tube. The suction tube was adjusted to maintain the fluid level in the flow channel at ∼2 mm. Oocytes were clamped at a holding potential of −60 mV using an OC-725C amplifier (Warner Instruments) connected by an LIH-1600 (HEKA) to a personal computer. Pulse 8.78 (https://www.heka.com/) software (HEKA) was used for data acquisition. ENaC-mediated whole-cell currents (ΔI_ami_) were determined by washing out amiloride (2 μM) with amiloride-free bath solution and subtracting the averaged whole-cell currents measured in the presence of amiloride at the beginning and the end of each recording from the corresponding whole-cell currents recorded in its absence. ND96 was used as a standard bath solution (composition in millimolar: 96 NaCl, 2 KCl, 1.8 CaCl_2_, 1 MgCl_2_, 5 Hepes; pH 7.4 adjusted with Tris).

### Molecular docking

The published structure of the ECL of human αβγ-ENaC (PDB-ID: 6WTH; (8, 71)) was prepared for docking by adding missing hydrogen atoms and residues and by capping open protein N- and C-termini with N-methyl amide and N-acetyl groups (ACE), respectively, by using Schrödinger Maestro Protein Preparation Wizard version 2021-2. In addition, the structure was relaxed using 300 ns atomistic MD simulations according to the protocol described below. The structure of S3969 was extracted from the ZINC database version 20 (ZINC-ID: ZINC43197820) (72). A receptor grid to define docking boundaries was generated using the Schrödinger Glide Docking module. Ten binding modes of S3969 were predicted for each of the two putative S3969-binding sites in β-ENaC using Glide Docking with the XP (extra precision) setting and flexible ligand sampling. After post-docking minimization of the S3969–ENaC complexes, docking results were visually inspected using Schrödinger Maestro. Binding modes were ranked according to the GlideScore value, which corresponds to the estimated S3969 binding free energy. The ENaC–S3969 complexes with the highest rank were chosen as starting configurations for the MD simulations described below.

### Atomistic MD simulations

Model systems with simulation box dimensions of approx. 114 × 114 × 114 Å were prepared using 3D-periodic boundaries with the Schrödinger Desmond module. Model systems contained approx. 120,000 atoms including atoms from αβγENaC ECLs (with or without S3969 in complex), water molecules (simple point charge model), sodium chloride (145 mM), and sodium counterions to achieve electroneutrality. MD simulations were conducted in the *NPT* ensemble imposing 1 atm and 310 K, respectively, with Schrödinger Desmond using the OPLS4 force field. Total simulation duration varied from 300 to 500 ns as indicated in the text or in the figure legends with MD trajectory sampling intervals of 100 ps. All dynamics simulations started with Desmond's default step-wise relaxation protocol for *NPT* ensembles over 160 ps. Subsequently, the temperature was kept constant at 310 K using the Nosé-Hoover thermostat with a relaxation time of 1 ps. In turn, isotropic pressure of 1.013 bar was maintained with the Martyna-Tobias-Klein barostat with a relaxation time of 2 ps. The cutoff radius for the Coulomb interactions was set to 12 Å. The integration timestep of a reversible reference system propagator algorithm integrator was set to 2, 2, and 6 fs for bonded, near, and far interactions, respectively. A positional restraint with a force constant of 100 kcal/mol/Å^2^ was applied to the capped protein termini. The conformational stability of ENaC during MD simulations was assessed by calculating RMSD from the starting structure. Critical interactions between ENaC and S3969 were identified using the Schrödinger Simulation Interactions Diagram tool. For each β-ENaC residue belonging to the α5 helix, the distance between its center of mass and the center of mass of the closest residue in γ-ENaC was calculated in each MD trajectory frame. For this purpose, we generated a Python script to operate with the analysis module of the Schrödinger Python Application Programming Interface (API). The script is available at https://github.com/f-sure/minimal-distance-MD. Structure visualizations were prepared using UCSF Chimera developed by the Resource for Biocomputing, Visualization, and Informatics at the University of California, San Francisco, with support from the National Institutes of Health P41-GM103311 (73). The molecular surface calculation was performed using the MSMS package with a probe radius of 1.4 Å and a vertex density of 2 vertices/Å^2^ (74).

### H441 cell culture experiments

The National Cancer Institute-H441 (H441) (American Type Culture Collection HTB-174) human lung epithelial cell line was obtained from American Type Culture Collection. Cells were maintained in a 5% CO_2_ atmosphere at 37 °C in H441 growth medium (RPMI1640 medium [Roswell Park Memorial Institute; Biochrom], 10% fetal bovine serum, 2 mM L-glutamine, 5 μg/ml apotransferrin, 5 μg/ml insulin, 10 nM sodium selenite, 1 mM sodium pyruvate, 100 U/ml penicillin, and 100 μg/ml streptomycin). For transepithelial measurements, cells (passages 68–70) were cultured on permeable supports (Millicell PCF membrane inserts; Merck-Millipore) in the H441 growth medium. At day 4 after seeding, this medium was replaced in the basolateral compartment by a differentiation H441 medium (RPMI1640 medium, 4% charcoal-stripped serum, 1 nM triiodothyronine, 50 nM dexamethasone, 5 μg/ml apotransferrin, 5 μg/ml insulin, and 10 nM sodium selenite, 100 U/ml penicillin, and 100 μg/ml streptomycin). The apical side of the epithelial monolayer was kept at an air–liquid interface. After 5 days at the air–liquid interface, the monolayers were transferred into Ussing chambers to measure the equivalent short circuit current (*I*_SC_) essentially as described previously (14, 64, 75). For these measurements, a Ringer's solution (composition in mM: 117 NaCl, 25 NaHCO_3_, 4.7 KCl, 1.2 MgSO_4_, 1.2 KH_2_PO_4_, 2.5 CaCl_2_, 11 D-glucose; equilibrated to pH 7.4 with a 5% CO_2_ atmosphere) was added to apical and basolateral compartments, and cells were allowed to equilibrate for 30 min. S3969 or amiloride were added directly to the apical bath solution from their respective stock solutions to achieve the final concentration of 10 μM.

### Statistical methods

N indicates the number of different batches of oocytes, and n indicates the number of individual oocytes studied per experimental group. Data are presented as mean ± SD. The normal distribution of data was assessed using the D'Agostino-Pearson omnibus test. Paired Student's *t* test or one-way ANOVA with Bonferroni’s post hoc test were used to assess statistical significance, as indicated in figure legends. Significant stimulatory effects of S3969 are indicated by asterisks (∗, ∗∗, or ∗∗∗) corresponding to *p* <0.05, *p* <0.01, or *p* <0.001, respectively.

To estimate half-maximal stimulatory concentration (EC_50_) of S3969, data of concentration-dependent stimulation of ENaC currents by S3969 were normalized, summarized, plotted on a log_10_ concentration scale, and fitted using the following equation:

(1) $${\Delta I}_{ami}=1+\left({\Delta I}_{ami,\max}-1\right)/\left[1+{\left(\frac{\log_{10}\left[C_{50}\right]}{\log_{10}\left[C\right]}\right)}^{Hill}\right]$$

where ${\Delta I}_{ami}$ is the actual normalized ENaC current, ${\Delta I}_{ami,\max}$ is the maximal normalized ENaC current reached at the highest concentrations of S3969 (10 μM), C is the actual S3969 concentration, $C_{50}$ is the S3969 concentration at which the half-maximal stimulation occurs (EC_50_), and Hill is the Hill coefficient. When the saturating concentration of S3969 was not reached, data points were fitted with a spline function, as indicated. Statistical analysis and graphical representation of results were performed using GraphPad Prism version 5.04 (GraphPad Software Inc) and R environment for statistical computation version 4.0.3 (https://www.R-project.org/).

## Data availability

All data are contained within the manuscript and the Supporting information. The python script used for the analysis presented in Figure 7 is available at https://github.com/f-sure/minimal-distance-MD.

## Supporting information

This article contains supporting information (8, 71).

## Conflict of interest

The authors declare that they have no conflicts of interest with the contents of this article.
