# Supplementary material for: The small molecule activator S3969 stimulates the epithelial sodium channel by interacting with a specific binding pocket in the channel's β-subunit
Source: J Biol Chem. 2024 Feb 23;300(4):105785. doi: 10.1016/j.jbc.2024.105785 (PMC11065748; doi:10.1016/j.jbc.2024.105785)
Supplement: Supporting Figures S1–S12 [file mmc1.pdf]

The small molecule activator S3969 stimulates the epithelial sodium channel (ENaC) by interacting with a specific binding pocket in the channel's β-subunit

Florian Sure[1], Jürgen Einsiedel[2], Peter Gmeiner[2], Patrick Duchstein[3], Dirk Zahn[3], Christoph Korbmacher[1], and Alexandr V. Ilyaskin[1*]

Supporting information Table of Contents

Figure S1; Figure S2; Figure S3; Figure S4; Figure S5; Figure S6; Figure S7; Figure S8; Figures S9; Figure S10; Figure S11; Figure S12.

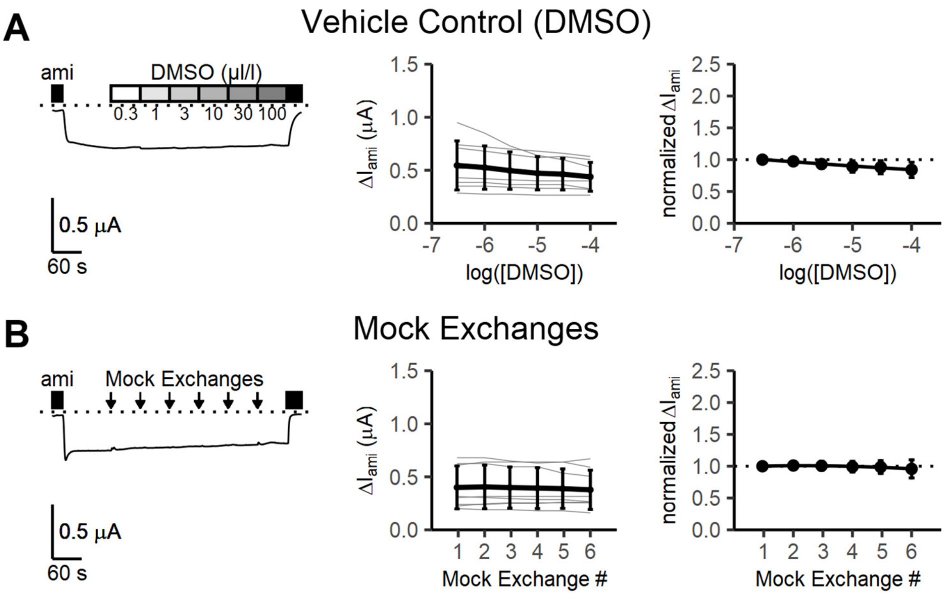

Figure S1: Vehicle control and mock solution exchange experiments.
A,B: *Left panels:* Representative whole-cell current traces obtained in individual oocytes expressing human $\alpha_h\beta_h\gamma_h$-ENaC. Amiloride (ami, 2 µM) and DMSO (at the indicated concentration in µl/l) were present in the bath solution as indicated by black and grey shaded bars, respectively. Arrows in *B* indicate time points at which mock bath solution exchanges occurred. Dotted lines correspond to the zero current level. *Middle panels*: ENaC-mediated amiloride-sensitive whole-cell current values (ΔI_{ami}) were determined from similar experiments as shown in the *left panel*. Grey lines connect data points obtained in an individual oocyte. Mean ± SD are shown in black (N=1, *n*=8; N indicates the number of different batches of oocytes, *n* indicates the number of individual oocytes studied per experimental group). *Right panels*: Concentration-response relationship of the DMSO effect on ENaC-currents (*A*) or time-course of ENaC-currents during the mock solution exchanges (*B*). In each individual recording shown in the *middle panel*, ΔI_{ami} was normalized to ΔI_{ami} obtained with 0.03 µl/l of DMSO (*A*) or to ΔI_{ami} reached during the first mock exchange (*B*). Mean ± SD are shown and fitted to a spline function. Dotted lines indicate normalized ΔI_{ami} values of one (no effect). One-way ANOVA with Bonferroni's post-hoc test was used to calculate p-values for comparisons with the baseline current values: in *middle panels* (*A*: 1 / 1 / 1 / 1 / 1 / 1; *B*: 1 / 1 / 1 / 1 / 1 / 1); in *right panels* (*A*: 1 / 1 / 0.083 / 0.004 / 0.002 / <0.0001; *B*: 1 / 1 / 1 / 1 / 1 / 0.903).

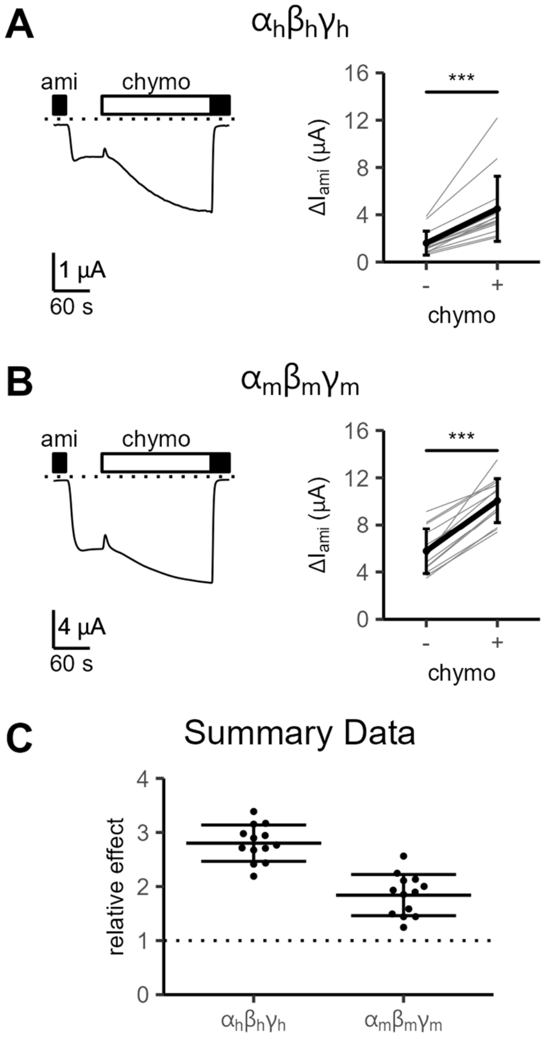

Figure S2: Human and murine ENaC can be activated by chymotrypsin.
A-B: *Left panels:* Representative whole-cell current traces obtained in individual oocytes expressing human ($\alpha_h\beta_h\gamma_h$, *A*) or mouse ENaC ($\alpha_m\beta_m\gamma_m$, *B*). Amiloride (ami, 2 µM) and chymotrypsin (chymo, 2 µg/ml) were present in the bath solution as indicated by black and white bars, respectively. Dotted lines correspond to the zero current level. *Right panels*: ENaC-mediated amiloride-sensitive whole-cell current values (ΔI_{ami}) were determined in the absence (-) or presence (+) of chymotrypsin from similar experiments as shown in the corresponding *left panel*. Grey lines connect data points obtained in an individual oocyte. Mean ± SD are shown in black (*A*: N=2, *n*=14; *B*: N=2, *n*=13; N indicates the number of different batches of oocytes, *n* indicates the number of individual oocytes studied per experimental group). Paired *t*-test was used to calculate p-values (A: <0.0001, B: <0.0001). ***p<0.001 indicates significant stimulation by chymotrypsin. *C:* relative stimulatory effect of chymotrypsin on ΔI_{ami} summarized from data shown in *A,B*. Dotted line indicates a normalized ΔI_{ami} value of 1 (no effect).

β-ENaC
sequence alignment

```
human    MHVKKYLLKGLHRLQKGPGYTYKELLVWYCDNTNTHGPKRIICEGPKKKAMWFLLTLLFA  60
mouse    MPVKKYLLKCLHRLQKGPGYTYKELLVWYCNNTNTHGPKRIICEGPKKKAMWFLLTLLFA  60
         * ******* ********************:*****************************

human    ALVCWQWGIFIRTYLSWEVSVSLSVGFKTMDFPAVTICNASPFKYSKIKHLLKDLDELME  120
mouse    CLVCWQWGVFIQTYLSWEVSVSLSMGFKTMNFPAVTVCNSSPFQYSKVKHLLKDLDELME  120
         .*******:**:************:*****:*****:**:***:***:************

human    AVLERILAPELSHANATRNLNFSIWNHTPLVLIDERNPHHPMVLDLFGDNHNGLTSSSAS  180
mouse    AVLEKILAPEASHSNTTRTLNFTIWNHTPLVLIDERNPDHPVVLNLFGDSHNSSN--PAP  178
         ****:***** **:*:**.***:***************.**:**:****.**. .   *

human    EKICNAHGCKMAMRLCSLNRTQCTFRNFTSATQALTEWYILQATNIFAQVPQQELVEMSY  240
mouse    GSTCNAQGCKVAMRLCSANGTVCTLRNFTSATQAVTEWYILQATNIFSQVLPQDLVGMGY  238
          . ***:***:****** * * **:*********:************:**  *:** *.*

human    PGEQMILACLFGAEPCNYRNFTSIFYPHYGNCYIFNWGMTEKALPSANPGTEFGLKLILD  300
mouse    APDRIILACLFGTEPCSHRNFTPIFYPDYGNCYIFNWGMTEETLPSANPGTEFGLKLILD  298
           :::*******:***.:**** ****.*************::*****************

human    IGQEDYVPFLASTAGVRLMLHEQRSYPFIRDEGIYAMSGTETSIGVLVDKLQRMGEPYSP  360
mouse    IGQEDYVPFLASTAGARLMLHEQRTYPFIREEGIYAMAGTETSIGVLVDKLQRKGEPYSP  358
         ***************.********:*****:******:*************** ******

human    CTVNGSEVPVQNFYSDYNTTYSIQACLRSCFQDHMIRNCNCGHYLYPLPRGEKYCNNRDF  420
mouse    CTMNGSDVAIKNLYSVYNTTYSIQACLHSCFQDHMIRNCSCGHYLYPLPEGEKYCNNRDF  418
         **:***:* ::*:** ***********:***********.*********.**********

human    PDWAHCYSDLQMSVAQRETCIGMCKESCNDTQYKMTISMADWPSEASEDWIFHVLSQERD  480
mouse    PDWAYCYLNLQMSVTQRETCLSMCKESCNDTQYKMTISMADWPSEASEDWILHVLSQERD  478
         ****:** :*****:*****:.*****************************:********

human    QSTNITLSRKGIVKLNIYFQEFNYRTIEESAANNIVWLLSNLGGQFGFWMGGSVLCLIEF  540
mouse    QSSNITLSRKGIVKLNIYFQEFNYRTIEESPANNIVWLLSNLGGQFGFWMGGSVLCLIEF  538
         **:*************************** *****************************

human    GEIIIDFVWITIIKLVALAKSLRQRRAQASYAGPPPTVAELVEAHTNFGFQPDTAPRSPN  600
mouse    GEIIIDFIWITIIKLVASCKGLRRRRPQAPYTGPPPTVAELVEAHTNFGFQPDTTSCRPH  598
         *******:********* .*.**:** ** *:**********************:   *:

human    TGPYPSEQALPIPGTPPPNYDSLRLQPLDVIESDSEGDAI 640
mouse    GEVYPDQQTLPIPGTPPPNYDSLRLQPLDTMESDSEVEAI 638
            **.:*:********************.:***** :**
```

palm β-ball finger GRIP thumb knuckle transmembrane intracellular

Figure S3: Sequence alignment of human and mouse β-ENaC
Symbols below the sequence indicate conserved sites (*), conservative replacements (:), semi-conservative replacements (.). Empty space ( ) denotes non-conservative replacements. Residues are colored according to β-ENaC domain organization as indicated.

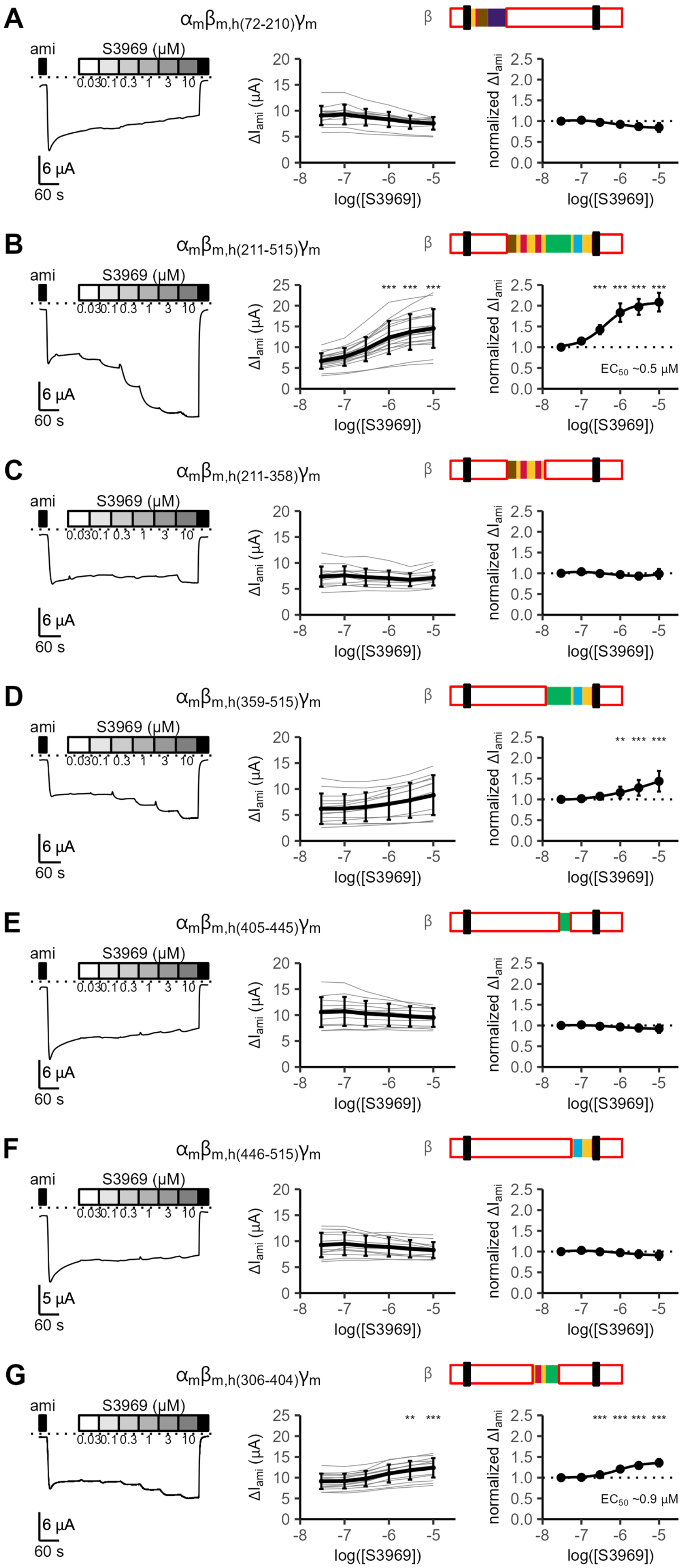

A
B
C
D
E
F
G
αmβm,h(72-210)γm
αmβm,h(211-515)γm
αmβm,h(211-358)γm
αmβm,h(359-515)γm
αmβm,h(405-445)γm
αmβm,h(446-515)γm
αmβm,h(306-404)γm
β
ami
S3969 (µM)
0.03 0.1 0.3 1 3 10
6 µA
5 µA
60 s
ΔIami (µA)
normalized ΔIami
log([S3969])
EC50 ~0.5 µM
EC50 ~0.9 µM

Figure S4: Identification of a β-ECL portion critically involved in the stimulatory effect of S3969

A-G: *Left panels* Representative whole-cell current traces obtained in individual oocytes expressing mouse α_m- and γ_m- subunits together with a chimeric mouse-human β subunit ($\beta_{m,h}$) as indicated. Pictograms illustrate which portion of mouse β-ENaC was replaced by the corresponding region of human β-ENaC. The explanation of the color-code is given in Figure 2. The experimental protocol was similar to that described in Figure 1. *Middle* and *right panels* Concentration-response relationships of the S3969 effect on absolute (*middle panel*) or normalized ΔI_{ami} (*right panel*). Summary data obtained from similar experiments as shown in corresponding *left panels* were analyzed as described in Figure 1. Mean ± SD are shown. In *right panels* mean values are fitted to a spline function (*A*: N=2, *n*=14; *C*: N=2, *n*=14; *D*: N=2, *n*=12; *E*: N=2, *n*=12; *F*: N=2, *n*=12; N indicates the number of different batches of oocytes, *n* indicates the number of individual oocytes studied per experimental group) or to Equation 1 (*B*: N=3, *n*=18; *G*: N=3, *n*=16). One-way ANOVA with Bonferroni's post-hoc test was used to calculate p-values for comparisons with the baseline current values: in *middle panels* (*A*: 1 / 1 / 1 / 1 / 0.272 / 0.100; *B*: 1 / 1 / 0.125 / <0.0001 / <0.0001 / <0.0001; *C*: 1 / 1 / 1 / 1 / 1 / 1; *D*: 1 / 1 / 1 / 1 / 1 / 0.470; *E*: 1 / 1 / 1 / 1 / 1 / 1; *F*: 1 / 1 / 1 / 1 / 1 / 1; *G*: 1 / 1 / 1 / 0.137 / 0.004 / 0.0002); in *right panels* (*A*: 1 / 1 / 0.190 / 0.0002 / <0.0001 / <0.0001; *B*: 1 / 0.098 / <0.0001 / <0.0001 / <0.0001 / <0.0001; *C*: 1 / 1 / 1 / 1 / 0.037 / 1; *D*: 1 / 1 / 0.587 / 0.003 / <0.0001 / <0.0001; *E*: 1 / 1 / 1 / 1 / 0.133 / 0.017; *F*: 1 / 1 / 1 / 0.998 / 0.041 / 0.002; *G*: 1 / 1 / 0.0004 / <0.0001 / <0.0001 / <0.0001). **p<0.01 and ***p<0.001 indicate significant stimulation by S3969.

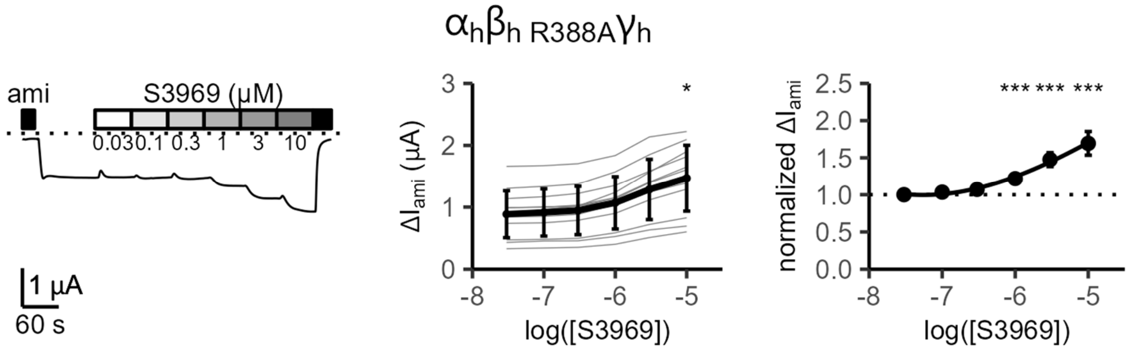

Figure S5: An arginine residue (Arg388) in β-ENaC plays an important role in channel stimulation by S3969

Representative whole-cell current trace (*left panel*) obtained in an oocyte expressing the mutant human ENaC ($\alpha_h\beta_{hR388A}\gamma_h$-ENaC) and summary data obtained from similar experiments showing concentration-response relationships of the stimulatory effect of S3969 on absolute (*middle panel*) or normalized ΔI_{ami} (*right panel*). Analysis was performed as described in Figure 1. Mean ± SD are shown. In the *right panel* mean values are fitted to a spline function (N=2, *n*=12; N indicates the number of different batches of oocytes, *n* indicates the number of individual oocytes studied per experimental group). One-way ANOVA with Bonferroni's post-hoc test was used to calculate p-values for comparisons with the baseline current values: in *middle panel* (1 / 1 / 1 / 1 / 0.561 / 0.032); in *right panel* (1 / 1 / 0.449 / <0.0001 / <0.0001 / <0.0001). *p<0.05 and ***p<0.001 indicate significant stimulation by S3969.

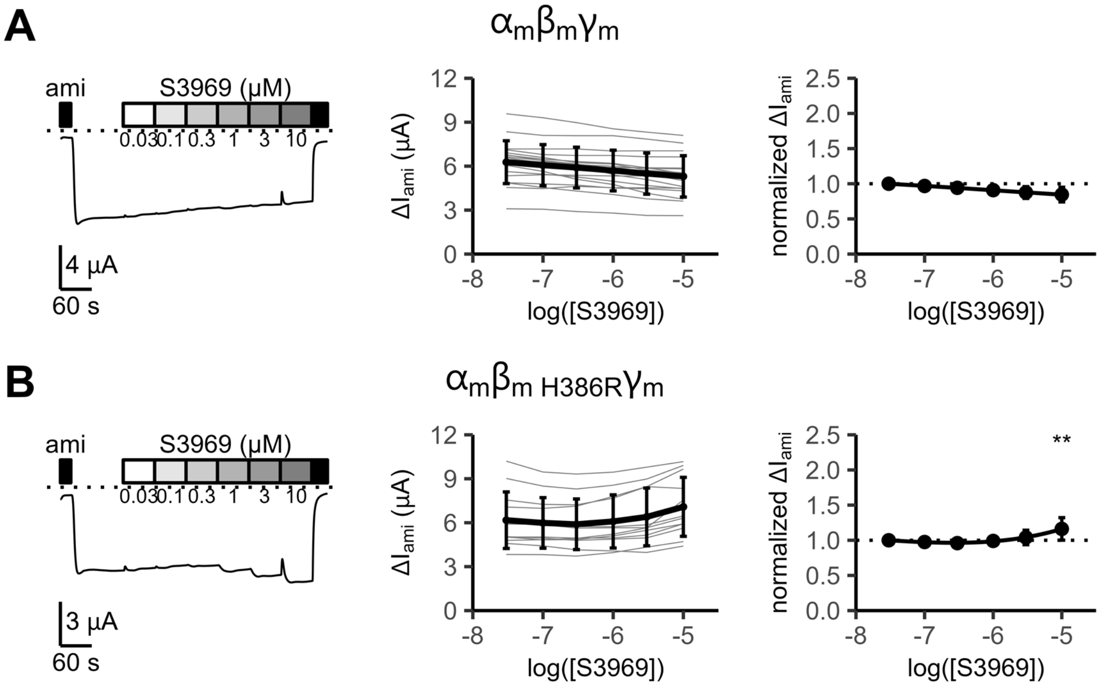

Figure S6: A histidine to arginine substitution (H386R) in the β-subunit converts mouse ENaC in a S3969-sensitive channel

Representative whole-cell current traces (*left panel*) obtained in individual oocytes expressing the wildtype ($\alpha_m\beta_m\gamma_m$-ENaC in *A*) or mutant mouse ENaC ($\alpha_m\beta_{m\ H386R}\gamma_m$-ENaC in *B*) and summary data obtained from similar experiments showing concentration-response relationships of the stimulatory effect of S3969 on absolute (*middle panel*) or normalized ΔI_{ami} (*right panel*). Analysis was performed as described in Figure 1. Mean ± SD are shown. In *right panels* mean values are fitted to a spline function (*A*: N=3, *n*=18; *B*: N=2, *n*=12; N indicates the number of different batches of oocytes, *n* indicates the number of individual oocytes studied per experimental group). One-way ANOVA with Bonferroni's post-hoc was used to calculate p-values for comparisons with the baseline current values: in *middle panels* (*A*: 1 / 1 / 1 / 1 / 0.978 / 0.368; *B*: 1 / 1 / 1 / 1 / 1 / 1); in *right panels* (*A*: 1 / 0.120 / 0.002 / <0.0001 / <0.0001/ <0.0001; *B*: 1 / 1 / 1 / 1 / 1 / 0.001). **p<0.01 indicates significant stimulation by S3969.

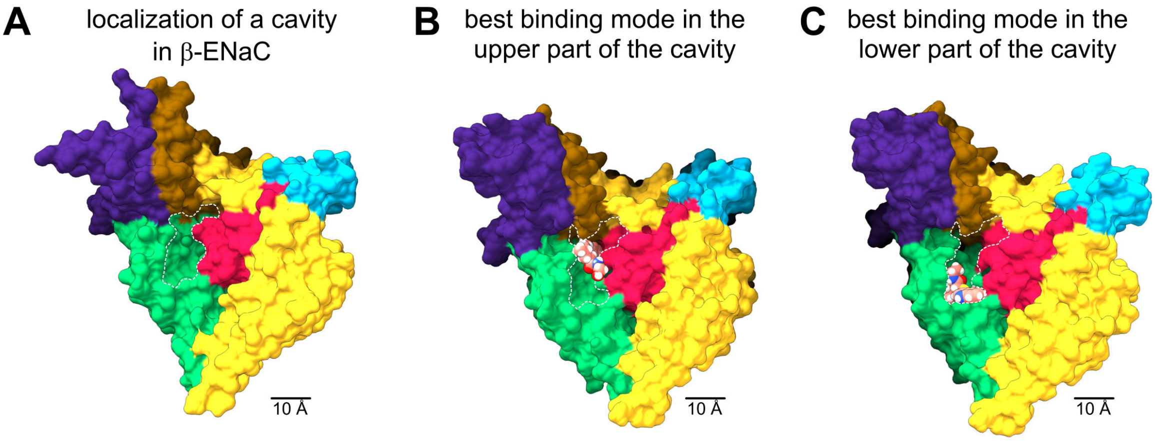

Figure S7: Prediction of a putative S3969 binding site in β-ENaC using molecular docking
A: Surface representation of the ECL of human β-ENaC generated using atom coordinates from PDB entry 6WTH (8,71). The color code is the same as in Figure 2. The localization of a cavity in the ECL of human β-ENaC is outlined with white dots. *B,C*: Results from the molecular docking approach using a modified structure of ENaC (see methods). The best S3969 binding mode in the upper (*B*) and the lower (*C*) part of the cavity. S3969 molecule is shown in sphere representation and colored using the same color code as in Figure 5.

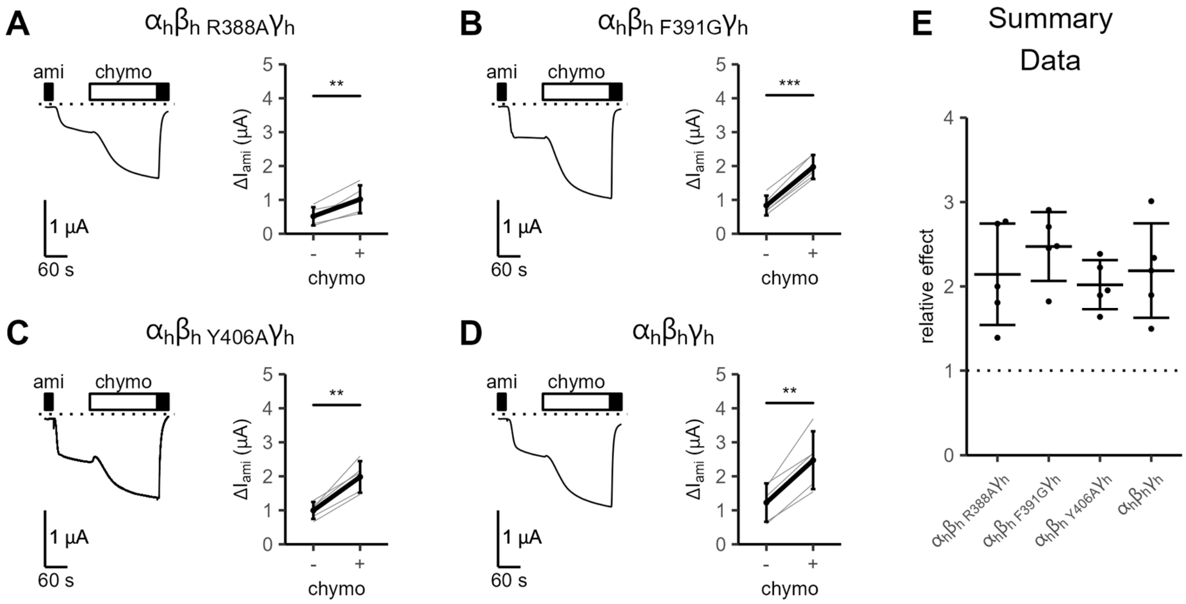

Figure S8: Human ENaC mutants with reduced responsiveness to S3969 can be activated by chymotrypsin in a similar manner as the wild-type channel.

A-D: Left panels: Representative whole-cell current traces obtained in individual oocytes expressing mutant (*A*: $\alpha_h\beta_{h\ R388A}\gamma_h$, *B*: $\alpha_h\beta_{h\ F391G}\gamma_h$, *C*: $\alpha_h\beta_{h\ Y406A}\gamma_h$), or wildtype human ENaC ($\alpha_h\beta_h\gamma_h$, *D*). Amiloride (ami, 2 µM) and chymotrypsin (chymo, 2 µg/ml) were present in the bath solution as indicated by black and white bars, respectively. Dotted lines correspond to the zero current level. *Right panels*: ENaC-mediated amiloride-sensitive whole-cell current values (ΔI_{ami}) were determined in the absence (-) or the presence (+) of chymotrypsin from similar experiments as shown in the corresponding *left panel*. Grey lines connect data points obtained in an individual oocyte. Mean ± SD are shown in black (N=1, *n*=5; N indicates the number of different batches of oocytes, *n* indicates the number of individual oocytes studied per experimental group). Paired *t*-test was used to calculate p-values (*A*: 0.010; *B*: <0.0001; *C*: 0.002, *D*: 0.004). **p<0.01 and ***p<0.001 indicate significant stimulation by chymotrypsin. *E:* relative stimulatory effect of chymotrypsin on ΔI_{ami} summarized from data shown in *A-D*. Dotted line indicates a normalized ΔI_{ami} value of 1 (no effect). One-way ANOVA with Bonferroni's post-hoc test indicates no significant difference in the chymotrypsin effects between mutant ENaCs and the wildtype (p-values are equal to 1 for all ENaC mutants).

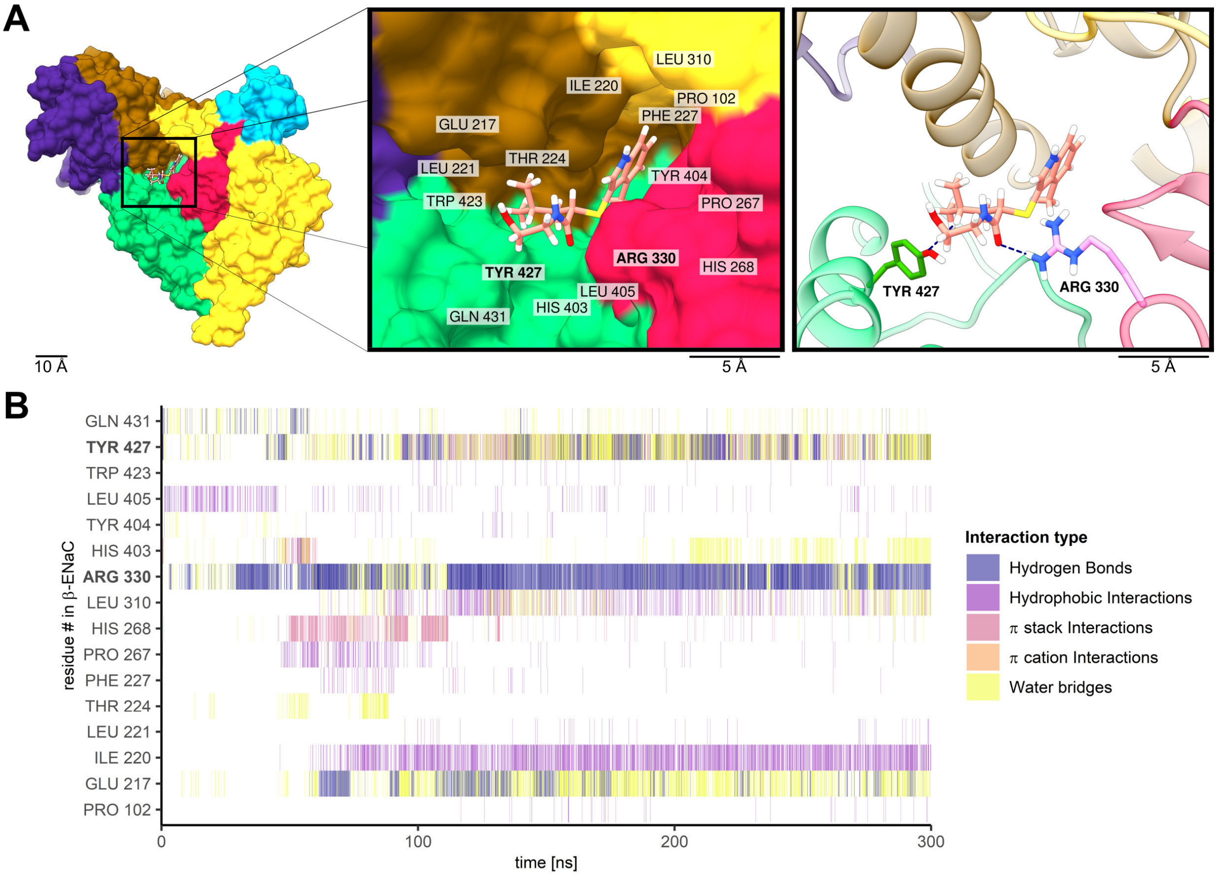

Figure S9: Atomistic molecular dynamics (MD) simulations of an S3969-ENaC complex, in which S3969 was bound to the upper part of the cavity in β-ENaC

A, Putative binding site of S3969 in the upper part of the cavity localized in the ECL of human β-ENaC. Snapshot is taken from the same MD simulation as in *B* at *t*=230 ns. The β-subunit is shown in surface representation and colored according to its domain organization using the same color code as in Figure 2. α- and γ-subunits are omitted for clarity. S3969 is shown in stick representation and colored using the same color code as in Figure 5. Insets show the S3969 binding site on an expanded scale in surface (*left inset*) or ribbon representation (*right inset*). In the *right inset*, Tyr427 and Arg330 residues forming stable interactions with S3969 are shown in stick representation with carbon atoms in green for Tyr427 and pink for Arg330, oxygen in red, nitrogen in blue and polar hydrogen in white. Apolar hydrogen atoms of β-ENaC are omitted for clarity. Blue dotted lines visualize hydrogen bond interactions. *B*, Diagram shows interactions formed between β-ENaC residues, which contribute to the putative S3969 binding site labeled in the *left inset* in *A* and an S3969 molecule during 300 ns MD simulations. Interaction types are represented by different colors as indicated. Each line represents the formation of at least one interaction of the specified type per trajectory frame. A darker color intensity indicates multiple interactions of the same type per trajectory frame.

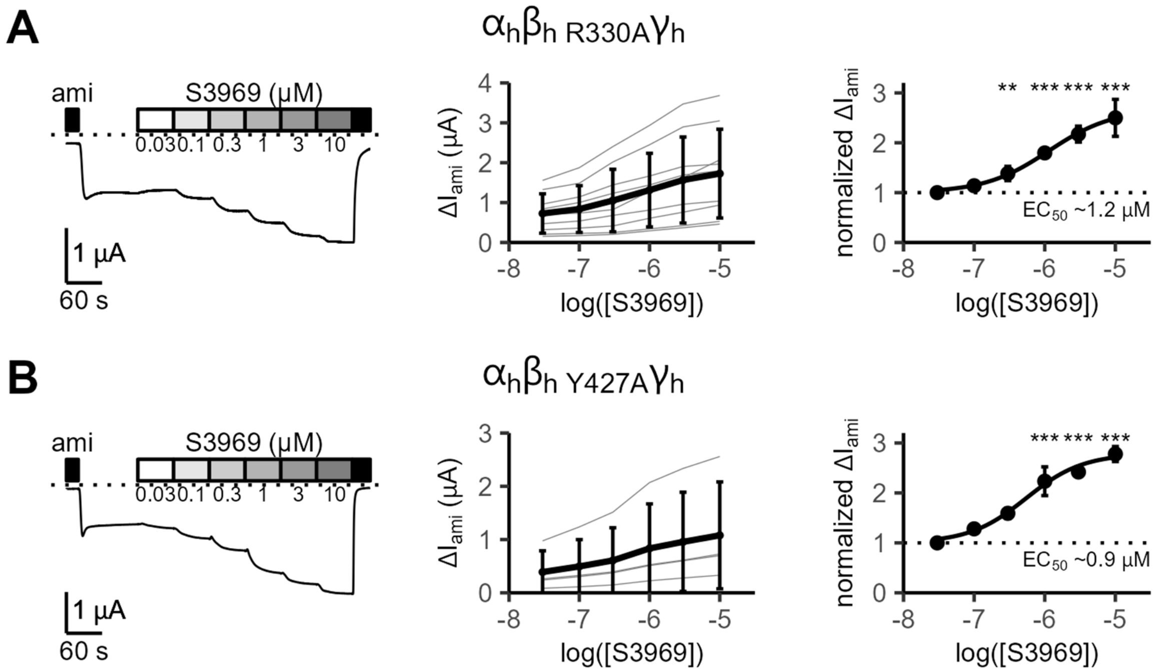

Figure S10: Mutations R330A and Y427A in the β-subunit have little effect on S3969-mediated ENaC activation

Representative whole-cell current traces (*left panels*) obtained in individual oocytes expressing $\alpha_h\beta_{h\ R330A}\gamma_h$-ENaC in *A* or $\alpha_h\beta_{h\ Y427A}\gamma_h$-ENaC in *B*, and summary data obtained from similar experiments showing concentration-response relationships of the stimulatory effect of S3969 on absolute (*middle panel*) or normalized ΔI_{ami} (*right panel*). Analysis was performed as described in Figure 1. Mean ± SD are shown. In *right panels* mean values are fitted to Equation 1 (*A*: N=2, *n*=9; *B*: N=1, *n*=4; N indicates the number of different batches of oocytes, *n* indicates the number of individual oocytes studied per experimental group). One-way ANOVA with Bonferroni's post-hoc test was used to calculate p-values for comparisons with the baseline current values: in *middle panels* (*A*: 1 / 1 / 1 / 1 / 0.910 / 0.337; *B*: 1 / 1 / 1 / 1 / 1 / 1); in *right panels* (*A*: 1 / 1 / 0.001 / <0.0001 / <0.0001 / <0.0001; *B*: 1 / 1 / 0.092 / <0.0001 / <0.0001 / <0.0001). **p<0.01 and ***p<0.001 indicate significant stimulation by S3969.

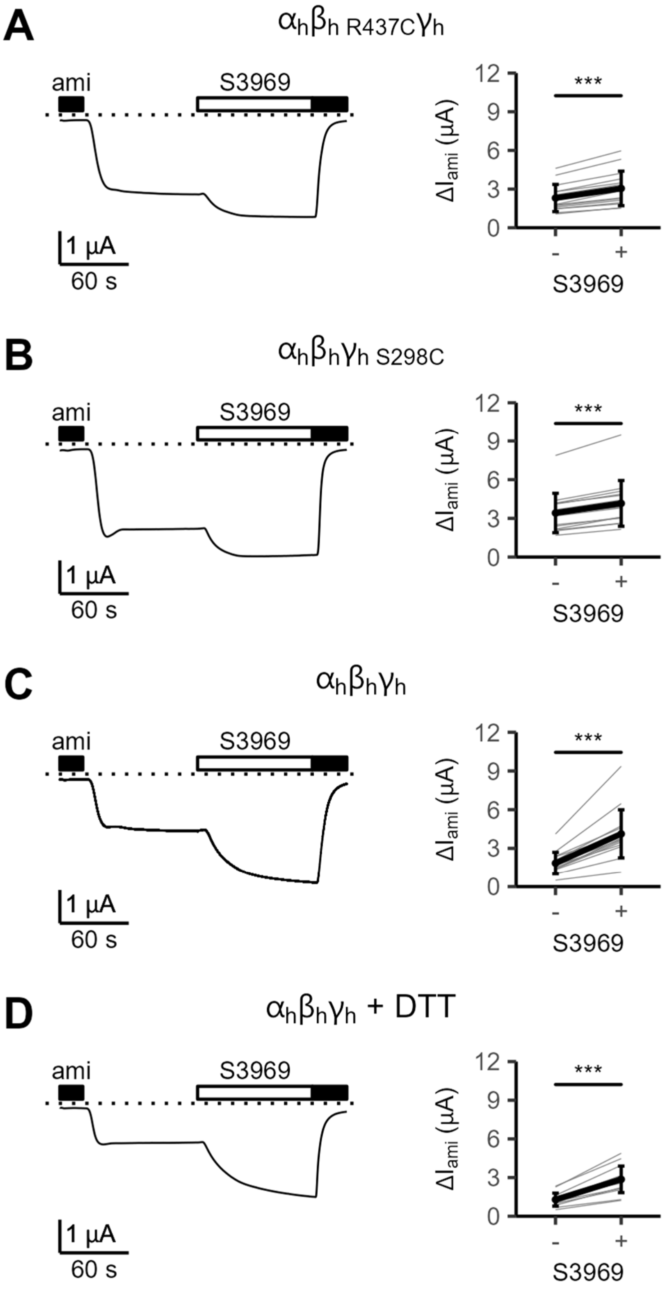

Figure S11: S3969-mediated ENaC stimulation in β- or γ-ENaC single cysteine mutants or in wildtype ENaC following DTT pre-incubation.
A-D: *Left panel* Representative whole-cell current traces are shown for individual oocytes expressing mutant human ENaC ($\alpha_h\beta_{h\ R437C}\gamma_h$ in *A*; $\alpha_h\beta_h\gamma_{h\ S298C}$ in *B*) or wildtype human ENaC ($\alpha_h\beta_h\gamma_h$ in *C,D*) without (*A-C*) or with (*D*) 15 min pre-incubation in 30 mM DTT. Amiloride (ami, 2 µM) and S3969 (10 µM) were present in the bath solution as indicated by black and white bars, respectively. Dotted lines correspond to the zero current level. *Right panels* Summary of ΔI_{ami} values measured before (−) and after (+) application of S3969 from similar experiments as shown in corresponding *left panels*. Grey lines connect data points obtained in an individual oocyte. Mean ± SD are shown in black (N=2, *n*=15; N indicates the number of different batches of oocytes, *n* indicates the number of individual oocytes studied per experimental group). Paired *t*-test was used to calculate p-values: (*A*: <0.0001, *B*: <0.0001, *C*: <0.0001, *D*: <0.0001). ***p<0.001 indicates significant stimulation by S3969.

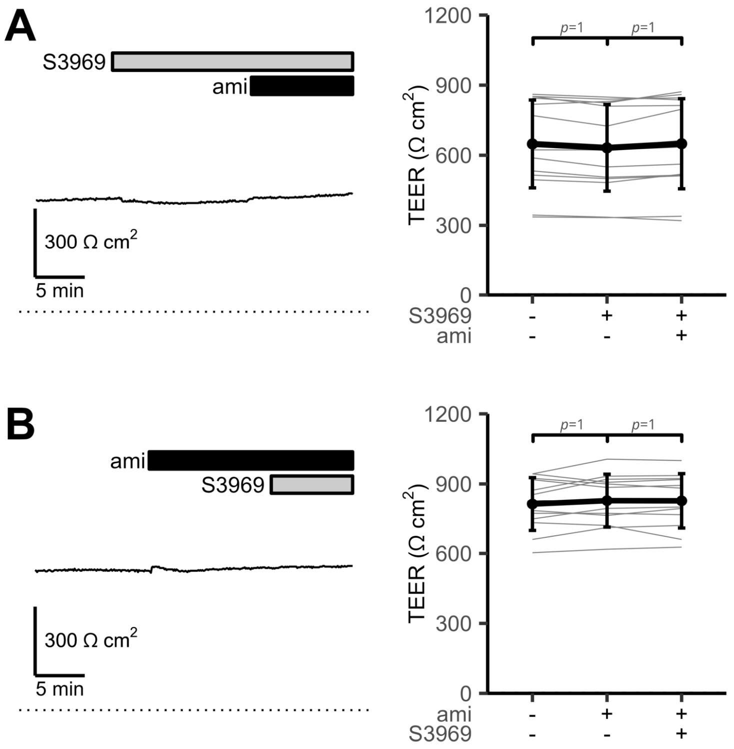

Figure S12: Application of S3969 did not significantly change the transepithelial resistance of H441 cell monolayers

A,B: Left panels: Representative transepithelial resistance (TEER) recordings, corresponding to I_{SC} traces shown in Figure 9, are shown. S3969 (10 µM) and amiloride (ami, 10 µM) were present in the apical bath solution as indicated by grey and black bars, respectively. Initial parts of recordings (~ 30 min) corresponding to the equilibration phase after transferring the cells into Ussing chambers and applying Ringer's solution to the apical compartment are omitted for clarity. The dotted lines indicate zero resistance level. *Right panels:* Summary data obtained in similar experiments as shown in *left panels*. TEER values were measured in each individual recording before the application of S3969 (*A*) or amiloride (*B*), before the subsequent application of amiloride in the presence of S3969 (*A*) or of S3969 in the presence of amiloride (*B*), and at the end of the experiment. Grey lines connect data points obtained from an individual H441 cell monolayer. Mean ± SD are shown in black (*A*: *n*=14; *B*: *n*=12). One-way ANOVA with Bonferroni's post-hoc test was used to calculate p-values.
